# Cardiovascular risk prediction models for women in the general population: A systematic review

**DOI:** 10.1371/journal.pone.0210329

**Published:** 2019-01-08

**Authors:** Sara J. Baart, Veerle Dam, Luuk J. J. Scheres, Johanna A. A. G. Damen, René Spijker, Ewoud Schuit, Thomas P. A. Debray, Bart C. J. M. Fauser, Eric Boersma, Karel G. M. Moons, Yvonne T. van der Schouw, on behalf of the CREW consortium

**Affiliations:** 1 Department of Cardiology, Erasmus Medical Center, Rotterdam, the Netherlands; 2 Netherlands Heart Institute, Utrecht, the Netherlands; 3 Julius Center for Health Sciences and Primary Care, University Medical Center Utrecht, Utrecht, the Netherlands; 4 Department of Clinical Epidemiology, Leiden University Medical Center, Leiden, the Netherlands; 5 Department of Vascular Medicine, Academic Medical Center, Amsterdam, the Netherlands; 6 Cochrane Netherlands, University Medical Center Utrecht, Utrecht University, the Netherlands; 7 Clinical Library, Academic Medical Center, Amsterdam, the Netherlands; 8 Department of Reproductive Medicine & Gynaecology, University Medical Center, Utrecht University, the Netherlands; University of Bologna, ITALY

## Abstract

**Aim:**

To provide a comprehensive overview of cardiovascular disease (CVD) risk prediction models for women and models that include female-specific predictors.

**Methods:**

We performed a systematic review of CVD risk prediction models for women in the general population by updating a previous review. We searched Medline and Embase up to July 2017 and included studies in which; (a) a new model was developed, (b) an existing model was validated, or (c) a predictor was added to an existing model.

**Results:**

A total of 285 prediction models for women have been developed, of these 160 (56%) were female-specific models, in which a separate model was developed solely in women and 125 (44%) were sex-predictor models. Out of the 160 female-specific models, 2 (1.3%) included one or more female-specific predictors (mostly reproductive risk factors). A total of 591 validations of sex-predictor or female-specific models were identified in 206 papers. Of these, 333 (56%) validations concerned nine models (five versions of Framingham, SCORE, Pooled Cohort Equations and QRISK). The median and pooled C statistics were comparable for sex-predictor and female-specific models. In 260 articles the added value of new predictors to an existing model was described, however in only 3 of these female-specific predictors (reproductive risk factors) were added.

**Conclusions:**

There is an abundance of models for women in the general population. Female-specific and sex-predictor models have similar predictors and performance. Female-specific predictors are rarely included. Further research is needed to assess the added value of female-specific predictors to CVD models for women and provide physicians with a well-performing prediction model for women.

## Introduction

Differences between women and men in cardiovascular disease (CVD) have been recognized decades ago [1], pertaining to clinical presentation, pathophysiological mechanisms, course of disease and prognosis [2–6]. As symptoms of CVD are more subtle in women, there is often delayed diagnosis, and thus treatment and consequently poorer prognosis and outcomes compared with men [7]. It is crucial to identify sex differences to optimize diagnostic and management strategies for both women and men [8]. Although women and men share many CVD risk factors, which are often used in prediction models for the general population, there are also female-specific risk factors. Well known examples are early menarche and menopause, primary ovarian insufficiency, pregnancy complications, polycystic ovary syndrome, and use of hormones [9–11].

Preventive measures are available to reduce the cardiovascular disease burden. Numerous strategies to reduce the CVD burden have been implemented to identify persons at high risk. As seen in a systematic review published in 2016, over 350 prediction models have been developed in recent years aiming to identify individuals at high CVD risk in the general population [12]. Guidelines in Europe and the Unites States currently recommend the use of Systematic COronary Risk Evaluation (SCORE) or the Pooled Cohort Equations in the general population, both for women and men [13,14].

Although several female-specific CVD risk factors have been identified, predictors in most implemented CVD prediction models seem generally similar for women and men. As clinical presentation, pathophysiological mechanisms, course of disease and prognosis differ between women and men; risk prediction likely differs between the sexes as well. Therefore, we aimed to provide an overview of available CVD risk prediction models for women and of models that include female-specific predictors.

## Methods

### Systematic literature search

For this review we used the results of the review by Damen *et al* on all future CVD prediction models for the general population, both men and women [12]. As shown by this review, the number of newly developed CVD prediction models grew excessively in recent years. For this reason, we complemented the results of Damen *et al*, by performing an update of their search. Details of the review by Damen *et al* were published previously [12]. In the original search, Medline and Embase were searched until June 1^st^ 2013 in order to identify articles on prediction models for the occurrence of CVD in the general population, published after 2004. Articles which dated before 2004 were subtracted from the review by Beswick *et al* [15]. Articles were included when they reported one or more multivariable (i.e. including at least 2 predictors) prediction models, tools or scores to predict future CVD in the general population (development papers), articles that investigated the added value of certain predictors (incremental value papers) and articles that validated existing models (validation papers). Table 1 provides an overview of the key terminology.

**Table 1:** Key definitions.

**Model developed for women**	A model developed for women, either separately for women (female-specific model) or where sex is incorporated as a predictor (sex-predictor model)
**Female-specific model**	A model developed in a dataset of women only, with a separate regression model or risk chart for women
**Sex-predictor model**	A model developed in a dataset of women and men, which uses sex as a predictor in the model
**Development**	When a new model is derived from a dataset
**Incremental value paper**	When one or more predictors are added to an existing model to study whether the performance of the model improves after adding the predictor(s)
**Validation paper**	When the performance of an existing model is verified in a different population
**Female-specific predictor**	A risk factor that is very clearly female specific such as: early menarche and menopause, primary ovarian insufficiency, pregnancy complications, and polycystic ovary syndrome
**Discrimination** *C statistic*	Indicates how well the model distinguishes between persons with an outcome event and persons without an outcome event, often depicted as the C statistic Measure of discrimination of the model and quantifies the area under the receiver operator curve (ROC). Ranges from 0.5 to 1.0, where 0.5 resembles a coin-toss and 1.0 is a perfect discrimination.

For the present systematic review, we updated the search of Damen *et al* until 26^th^ of July 2017. Title and abstract screening were conducted using the same in- and exclusion criteria as Damen *et al*. However, in the full text screening we included only models specifically developed to predict CVD in women. We defined ‘model developed for women’ as 1) female-specific models, in which a separate model was developed in women only and 2) sex-predictor models, in which sex was included as a predictor (e.g. covariate) in the model (Table 1). Models that were developed on men only or models that did not include sex as a predictor were excluded. For the validation papers, only studies that validated a prediction model developed for women were included. Studies in which a predictor was added to an existing model (incremental value papers) were also included. Incremental value or validation studies in men only were excluded.

### Screening and data extraction

The titles and abstracts retrieved by the search were divided randomly among the researchers (SJB, VD or LJJS) and screened independently. Studies were not screened in duplicate, but to guarantee uniformity in screening, 30 abstracts were screened by all three researchers and discussed afterwards. In the screening stage, all papers that were labeled as ‘any doubt’ were included for full text screening.

For full text screening the papers were divided in three different subsets for independent screening by one of the three researchers (SJB, VD or LJJS). Again, full text screening was not performed in duplicate, a subset of 20 papers from each researcher was screened by all three researchers to achieve uniformity. Articles labeled as “any doubt” were resolved by discussion among the three reviewers to reach consensus. Hand searching based on included articles and 'snowballing' were used to search for additional studies.

Finally, data extraction was performed in a pre-specified data-extraction format based on the CHecklist for critical Appraisal and data extraction for systematic Reviews of prediction Modelling Studies (CHARMS) [16]. All three reviewers read the papers and subsequently filled in the data-extraction format together to guarantee agreement on the extracted information. In this stage, disagreements were settled by an additional reviewer (JAAGD or YTvdS). For papers in which a model was developed we extracted the same information as Damen *et al*. and additionally determined whether the model was a female-specific or sex-predictor model. All developed models were then assessed for quality based on reliability defined as 1) model externally validated 2) model externally validated in a separate investigation/paper and 3) C statistic > 0.7. If the development model did not report a C statistic, we used the mean C statistic of the external validations. Reliable models, which met these criteria were assessed for clinical usability for 1) 10 predictors or fewer, 2) full regression model or chart reported and 3) availability of an online calculator.

For every included incremental value paper we extracted author, year, journal, the model that was used to calculate incremental value and whether this model was female-specific or sex-predictor and which predictors actually had incremental value. In addition, predictors considered for incremental value were also extracted.

Finally, for the validation papers we extracted author, year, journal and which model was validated. For the models that were validated >5 times and at least once in an external study, we subsequently extracted additional information: characteristics of the validation cohort (country, number of participants, age range, number of events), and performance measures (Table 1). We also extracted whether the validation cohort existed of men and women or women only (studies with men only were previously excluded). When studies used a cohort consisting of both men and women, the model could be validated on men and women together or separately. When validated in men and women separately we only included the validation on women.

### Descriptive analyses

Results are presented as counts or percentages where indicated. Combined summary measures of studies and models (e.g. C statistics and number of participants in a cohort) are presented as medians and/or ranges. Proportions were compared with the Chi-square test. C statistics of the most frequently validated models were pooled with the R package metamisc [17]. We estimated random-effect models using restricted maximum likelihood estimation, and derived approximate 95% prediction intervals using the methods described in metamisc [17]. Analyses were performed using SPSS 24 (IBM, Armonk, New York) or R (R Core Team, Vienna, Austria).

## Results

**Fig 1:**
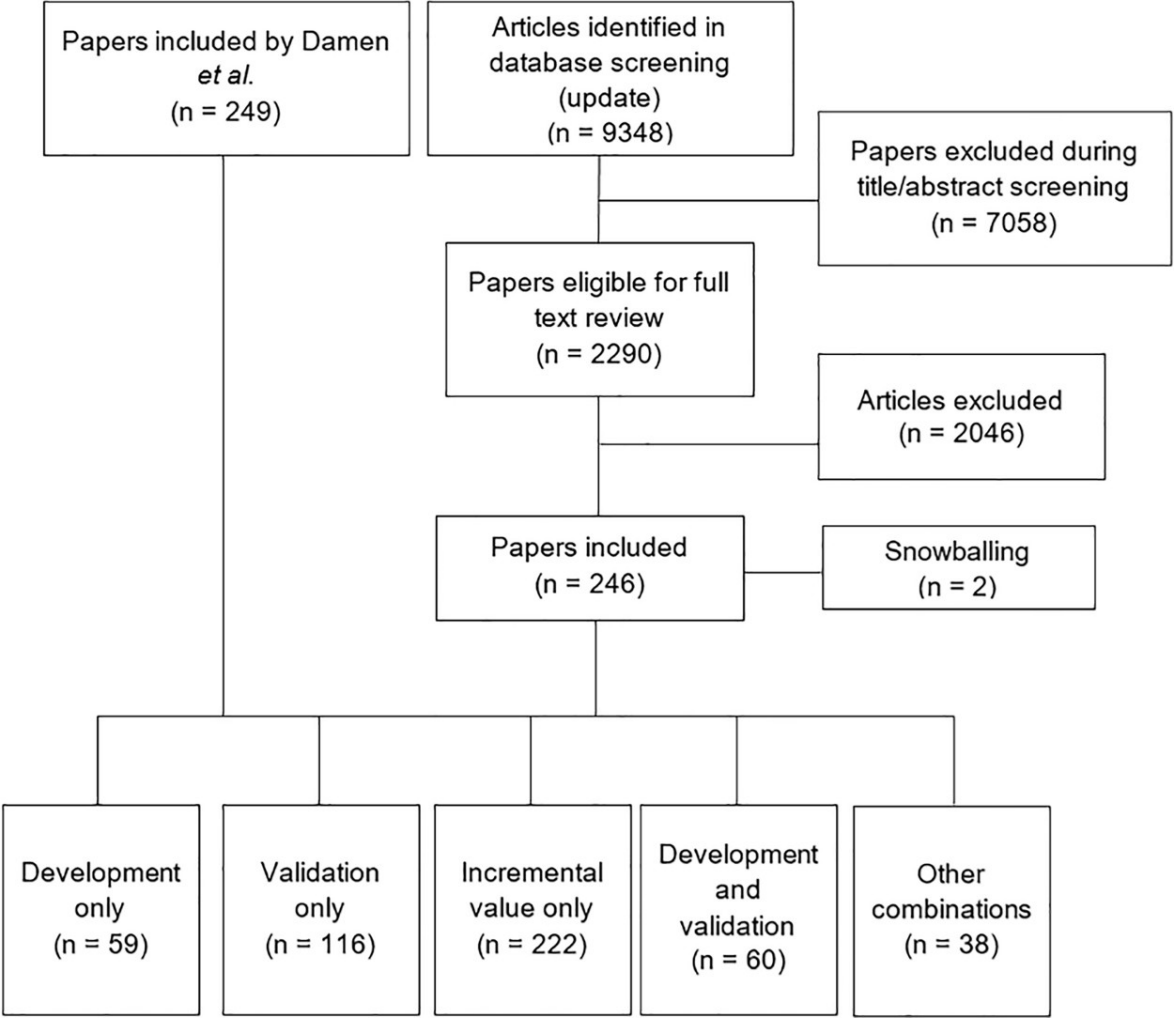
Study flow diagram. The papers that were identified by the updated search were added to the papers from the study by Damen and colleagues, resulting in a total of 495 papers.

Fig 1 depicts the study flow diagram. From the study by Damen and colleagues, 249 articles were included that described models developed for women. The updated search, after removing duplicates, resulted in 9348 new references. After title and abstract screening, 2290 articles were eligible for full text assessment. Full text screening resulted in 244 included articles from the updated search and two additional references identified through snowballing. These 246 papers were added to the 249 papers from Damen *et al* and in total, this review includes 495 papers on models for women (Fig 1).

In 133 papers prediction models for women were developed. In 206 papers a model was validated and 260 papers concerned incremental value studies. Since papers can develop a model, validate a model and calculate the incremental value of a predictor on an existing model in the same paper, these numbers do not add up to the total of 495 papers.

### Development of new prediction models

In 133 distinct papers, 285 cardiovascular risk prediction models were developed. Of these, 160 (56%) were developed solely on women and are henceforth denoted as female-specific models. The remaining 125 (44%) were sex-predictor models (Table 2). Table 2 shows the year in which the models were published. Clearly, new models are still being developed in large numbers, with the majority of the models developed in the last decade (on average 16 new models developed each year). Before 1990, 62% of the developed models were sex-predictor models. Between 1991 and 2010 female-specific models were developed more often than sex-predictor models, since 2010 these proportions are equally divided.

**Table 2:** Number of developed models over time.

Year	1967–1990	1991–2000	2001–2010	2011–2017	Total
**Sex predictor**	21 (62%)	21 (35%)	28 (35%)	55 (50%)	125 (44%)
**Female specific**	13 (38%)	39 (65%)	52 (65%)	56 (50%)	160 (56%)
**Total**	34 (100%)	60 (100%)	80 (100%)	111 (100%)	285 (100%)

### Predictors in the development papers

For the models that were specifically developed for women, it was of particular interest whether female-specific predictors were included in the model. Only 2 out of the 160 developed female-specific models (1.3%) included a female-specific risk factor. In the first, D’Agostino and colleagues developed a model including menopause (yes/no) and an interaction with menopause and age as predictors [18]. In the second, Parikh and colleagues considered the predictors pregnancy status, number of live births, age at menarche, menstrual irregularity, age at first birth, stillbirths, miscarriages, infertility ≥1 year, infertility cause and breastfeeding for inclusion in a model with established risk factors. The final model presented included in addition to age the female-specific risk factors: menstrual irregularity, age at first birth, still births, miscarriages and breastfeeding and had a C statistic of 0.675 in the derivation cohort [19].

**Fig 2:**
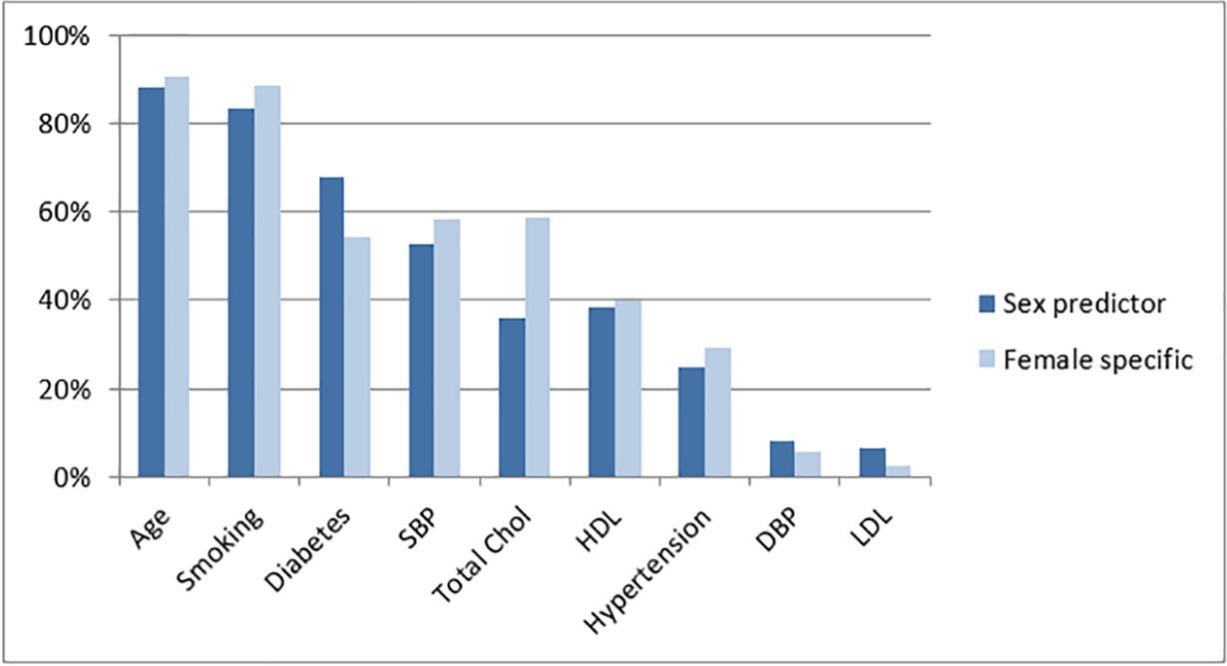
Most frequently used predictors for the sex predictor and female-specific models. HDL; High-density lipoprotein. Total Chol; total cholesterol. LDL; Low-density lipoprotein. SBP; systolic blood pressure. DBP; Diastolic blood pressure.

The median number of predictors for the female-specific models was 6 [IQR: 5–8] and for the sex-predictor models was 8 [IQR: 7–10], including the predictor for sex. Fig 2 shows the percentage of sex-predictor and female-specific models that included the nine most often-used predictors. By definition sex was not a predictor in any of the female-specific models. Total cholesterol was used more frequently in female-specific models (58% vs. 36%, difference 22% 95%CI 10%-33%). For the remaining eight predictors most frequently identified in the models (age, smoking, diabetes mellitus, systolic blood pressure, HDL, hypertension, diastolic blood pressure, and LDL), the frequency of predictors used was similar for the both model types.

The apparent C statistic (i.e. the C statistic in the development models) was reported in 66 (53%) of the sex-predictor models and in 59 (37%) of the female-specific models. The median of the C statistics were similar (0.797 for the sex-predictor models [range: 0.610–1.000] and 0.787 for the female-specific models [range: 0.660–0.918]). The full list of identified development papers in the updated search is available as S1 Table.

### Validation of prediction models

A total of 206 articles described 591 validations of sex-predictor or female-specific models. The models that were validated more than five times and at least once in a separate paper, were; SCORE Conroy 2003 (n = 63), Framingham Wilson 1998 (n = 61 validations), Pooled Cohort Equations Goff 2013 (n = 52), Framingham D'Agostino 2008 (n = 48), Framingham Anderson 1991a (n = 40), Framingham ATP III 2002 (n = 29), Framingham Wolf 1991 (n = 20), Framingham Anderson 1991b (n = 14), and QRISK Hippisley-Cox 2007 (n = 6) (Table 3). The 333 validations of these nine models will be discussed further. The only model that is a sex-predictor model is Framingham Anderson 1991a, which was validated 15 (37%) times in men and women and 25 (63%) times in women only. The eight female-specific models were validated 119 (41%) times in men and women together. The other 174 validations (59%) were performed in women only. A C statistic was reported in 70% of these validation studies and ranged from 0.449 to 0.993. Pooled C statistics showed similar performances in validations performed on women only and validations on men and women together (Table 4). The full list of validated models identified in the updated search is available as S2 Table.

**Table 3:** Characteristics of the validations of the nine most frequently validated prediction models.

	1	2	3	4	5	6	7	8	9
	SCORE	Framingham	Pooled Cohort Equations	Framingham	Framingham	Framingham	Framingham	Framingham	QRISK
	Conroy 2003	Wilson 1998	Goff 2013	D'Agostino 2008	Anderson 1991	ATP III 2002	Wolf 1991	Anderson 1991	Hippisley-Cox 2007
	n = 63	n = 61	n = 52	n = 48	n = 40	n = 29	n = 20	n = 14	n = 6
**Composition of validation cohorts**									
Men and Women	26	27	16	28	15	15	6	1	0
Women Separately	37	34	36	20	25	14	14	13	6
**Location of the validation cohorts**									
Asia	8	7	8	10	1	1	1	1	0
Australia	4	0	1	1	10	1	0	1	0
Europe	43	20	7	22	28	3	9	8	6
North America	8	32	34	13	1	24	10	4	0
**Participant age in the validation cohorts**									
Min, median	40	40	40	40	35	45	55	35	35
Max, median	65	74	79	79	74	82	99	64	74
**Size of the validation cohorts**									
Sample size, median [range]	7573 [203–44649]	3554 [246–163627]	4218 [392–307591]	2613 [136–542987]	2105 [302–797373]	3716 [613–36517]	3507 [401–23983]	3014 [331–542783]	542987 [306111–797373]
Events, median [range]	157 [10–4842]	213 [8–24659]	150 [9–4658]	146 [15–18173]	86 [1–29057]	384 [35–2343]	160 [24–939]	158 [5–18173]	29057 [18027–29057]

**Table 4:** Pooled C statistics of the validations of the nine most frequently validated prediction models.

	1	2	3	4	5	6	7	8	9
	SCORE	Framingham	Pooled Cohort Equations	Framingham	Framingham	Framingham	Framingham	Framingham	QRISK
	Conroy 2003	Wilson 1998	Goff 2013	D'Agostino 2008	Anderson 1991	ATP III 2002	Wolf 1991	Anderson 1991	Hippisley-Cox 2007
**Validations on men and women**									
	**n = 15**	**n = 17**	**n = 9**	**n = 21**	**n = 8**	**n = 11**	**n = 3**	**n = 0**	**n = 0**
Pooled C statistic	0.768	0.717	0.739	0.734	0.673	0.72	0.653	—	—
95% Prediction Interval	(0.709–0.826)	(0.542–0.893)	(0.679–0.799)	(0.600–0.868)	—^a^	(0.593–0.846)	—^a^	—	—
**Validations on women separately**									
	**n = 13**	**n = 18**	**n = 28**	**n = 10**	**n = 13**	**n = 8**	**n = 8**	**n = 2**	**n = 3**
Pooled C statistic	0.772	0.682	0.757	0.730	0.776	0.687	0.678	0.767	0.796
95% Prediction Interval	(0.591–0.954)	(0.491–0.874)	(0.696–0.819)	(0.544–0.916)	(0.755–0.796)	(0.568–0.806)	(0.447–0.908)	—^b^	(0.750–0.843)

a Due to limited information the resulting prediction interval lies outside the possible interval (values >1 and/or <0)

b Not enough validations were available to calculate the prediction interval

### Incremental value

In 260 articles the added value of a predictor to an existing female-specific or sex-predictor model was described. In 3 (1.1%) papers female-specific risk factors were added to an existing model, all of which were recently published (2016 n = 2 and 2017 n = 1) [20–22]. In the previously discussed paper by Parikh and colleagues, female-specific predictors were added to established risk factors, resulting in a final model including age at first birth, still births, miscarriages and breastfeeding. This slightly improved the model, C statistic of 0.730, where the model with only established risk factors had a C statistic of 0.726 [19]. In a study by van der Meer and colleagues, the female-specific predictors age at menarche, menopausal status/age, hormone use, gestational hypertension and diabetes, number of children, miscarriages/stillbirths were added to established risk factors. The addition of these predictors did non apparently improve the discrimination or calibration of the model beyond the established risk factors [23]. In the third paper, Zhou and colleagues added amongst other predictors (African American ethnicity, physical exercise level, BMI, waist circumference, height, HDL cholesterol), use of hormone replacement therapy in postmenopausal women to the Framingham Stroke Risk Score (Wolf 1991). The addition of this predictor set improved discrimination and calibration of the model in women; however, the separate performance of hormone use was not reported [24]. The full list of incremental value papers identified by the updated search is available as S3 Table.

### Reliability and clinical usability of available models

All 285 models developed for women were first assessed for reliability and were regarded so if they met the following criteria: 1) model externally validated 2) externally validated in a separate investigation/paper and 3) a C statistic >0.7. Of the 285 models, 40 (14%) met these criteria and were considered reliable (Table 5). Of these 40, 25 (63%) were female-specific and 15 (37%) were sex-predictor models. Following, these models were assessed for clinical usability based on the presence of 1) 10 predictors or fewer, 2) full regression model or chart reported and 3) online calculator available (Table 5). The SCORE and Framingham 2008 model had the highest usability score as they met all criteria. Other models with high usability are the Pooled Cohort Equations (African American), Framingham 30 year and the Framingham stroke models as they have 10 or fewer predictors and an online calculator available. The remaining models either had more than 10 predictors or no calculator available, rendering them less appealing for clinical practice.

**Table 5:** Clinical usability of models that met the reliability criteria.

Model–study name	Author—Year	Number of separate models	< 10 predictors	Full regression formula	Risk Chart	Online calculator
Framingham	Anderson 1991a	12	✓	✓	✕	✕
Framingham	Anderson 1991b	2	✓	✓	✓	✕
—	Assmann 2007	2	✓	✕	✓	✕
ARIC	Chambless 2003	2	✕	✓	✕	✓
SCORE	Conroy 2003	6	✓	✓	✓	✓
Framingham	D'Agostino 2008	2	✓	✓	✓	✓
Framingham	ATP II	1	✓	✓	✓	✕
—	Gaziano 2008	2	✓	✕	✓	✕
Pooled Cohort Equations (African American)	Goff 2013	1	✓	✓	✕	✓
Pooled Cohort Equations (White)	Goff 2013	1	✕	✓	✕	✓
QRISK	Hippisley-Cox 2007	1	✓	✕	✕	✕
QRISK2	Hippisley-Cox 2008	2	✕	✕	✕	✕
QRISK lifetime	Hippisley-Cox 2010	1	✕	✕	✕	✓
—	Lumley 2002	1	✓	✕	✓	✕
Framingham (30 yrs)	Pencina 2009	1	✓	✕	✕	✓
—	Schnabel 2009	1	✓	✕	✓	✕
Framingham	Wilson 1998	1	✓	✓	✓	✕
Framingham (Stroke)	Wolf 1991	1	✓	✕	✓	✓

Clinical usability was scored for the models which met all criteria for reliability: 1) model externally validated 2) externally validated in a separate investigation/paper and 3) a C statistic >0.7.

## Discussion

In this study we provided an overview of the available CVD risk assessment models for women in the general population. We identified a wide range of models that have been developed over the past decades, including 160 female-specific models (i.e. models that are developed for use in women only) and 125 sex-predictor models (i.e. models that include sex as a predictor). Despite this large quantity, only two of the 160 (1.3%) female-specific models included female-specific predictors [18,19]. Of the 260 studies in which the added value of a predictor was assessed, only three (1.1%) investigated the added value of a female-specific predictor [19,23,24].

Our study has several major strengths. We performed an extensive search up to July 2017 and systematically selected studies for inclusion. Detailed and thorough data extraction of essential information such as type of models, predictors, population and model discrimination, was performed by means of standardized forms and was done by three investigators together for the development models to ensure uniformity. Limitations of our study should be mentioned. First, we did not include models specifically made for men and thus could not compare differences in performance and predictors between men and women. Second, in some validation studies it was not clear which models were validated when the original development article reported on more than one model. We assumed that all models in the article were validated, but this may have led to an overestimation of the actual number of times prediction models were validated. Third, we did not include articles written in a language different than English and articles of which the full text could not be retrieved. Furthermore, validation papers were excluded from the pooled C statistic analyses when insufficient information necessary for pooling was reported. In addition, since we did not conduct a formal risk of bias assessment, we were only able to include all validation studies in which reporting was complete, instead of including for example studies with the smallest risk of bias. Therefore, results on the pooled C statistics, should be interpreted with caution. Finally, as calibration was reported in a heterogeneous manner, conclusions for this performance measure could not be drawn. Furthermore, in papers the measure for calibration was often not reported. In order to guarantee uniformity, new studies reporting on prediction models should adhere to the Transparent Reporting of a multivariable prediction model for Individual Prognosis Or Diagnosis (TRIPOD) statement [25,26].

The models described in this review often comprise several variations of established, sex-independent predictors such as age, blood pressure, lipid levels and smoking indicating that these predictors attribute most to the current performance of the models. Interestingly, the results showed that both the female-specific models as well as the sex-predictor models often comprise these same established predictors and do not differ substantially in estimated C statistic. This might imply that using sex as predictor in a model is just as effective as developing a female-specific model. Of the nine most frequently validated models in women the C statistic as a measure of performance was reported in 59% of the validation studies. Pooled C statistics indicated good performance in general (pooled C statistic >0.70 for most models), although the range of reported C statistics varied from 0.45 to 0.99. This indicates that although these models generally perform well, they can definitely be improved. Of all 285 developed models, only 40 (14%) met the quality criteria for reliability. When these models were further assessed for clinical usability only 2/40 (5%), the SCORE and Framingham 2008 model, met all criteria. Other models which met most criteria and had a risk calculator available were the Pooled cohort equations, Framingham 30 years and Framingham stroke model. Based on both these reliability and clinical usability criteria, these models seem best suitable for implementation in clinical practice. Models without an online calculator are likely less attractive for use in clinical practice.

Our findings are in line with a previous study by Goh and colleagues, in which the utility of CVD prediction models for women was appraised [20]. They also concluded that there is room for improvement in CVD prediction models for women and this could be achieved by adding predictors which may perform well in women. Remarkable is that none of the predictors suggested by Goh, such as obesity, physical activity and coronary artery calcium, are female-specific. It must be noted that in the study by Goh and colleagues the search was limited to five years before publication (2008–2013). The study was restricted to six models, where we in our study considered any model identified by the search strategy. The 2011 guidelines for the prevention of CVD in women [27] categorize women as ‘at risk’ when having one or more major risk factors. Aside from the established risk factors found in most prediction models, they explicitly include the female-specific risk factors of a history of preeclampsia, gestational diabetes, or pregnancy-induced hypertension. However, none of these disorders are used in any of the prediction models for women in this review.

Although many models have been developed in women only, it seems that differences between men and women in CVD risk assessment are still not fully recognized. Many female specific risk factors for CVD have been identified in recent years, but their predictive potential has not been tested or even considered in risk prediction models within the scope of our review. Our search only identified two development studies that included a female-specific predictor in the model [18,19]. Improvement of the existing models might be achieved in adding female-specific predictors. However, in most of the incremental value studies we found, female-specific predictors were not even considered as potential predictors for added value. Of the 260 incremental value studies, three added a female-specific predictor. Of these, one reported no improvement in performance and one observed a slight improvement in discrimination. The third did not report on improvement of individual predictors. A reason for not finding any substantial improvement could be that studies missed information on several important female-specific risk factors like preeclampsia, polycystic ovary syndrome and infant birth weight. Therefore, it is important to further investigate the potential added value of female-specific predictors. Most female-specific predictors become apparent at an early stage in life whereas CVD events mostly occur after the age of 50. An additional benefit is that these predictors can be easily obtained from the medical history. This underlines the potential of these predictors, as risk assessment is ideally performed decades before the anticipated event, in order to implement and optimize effect of preventive strategies. Although we identified a total of 495 papers on CVD prediction models for women, it is still uncertain whether these can be improved by female-specific predictors. However, it should be mentioned that finding new predictors that improve model performance on top of the well-known predictors seems challenging [22]. It is possible that current models, which often aim to estimate the 10-year risk based on a single assessment, have reached their maximal predictive potential and cannot be further improved. A new type of model, for example the dynamic model, in which an individual’s risk is continuously updated over time, could further advance preventive strategies.

## Conclusions

In conclusion, there is an abundance of models for women in the general population, but female-specific predictors are rarely included. The few studies that add female-specific risk factors to existing CVD risk models do not show substantial improved performance, but lacked important potential predictors. Further research in order to provide physicians with a well-performing and properly validated prediction model for women is therefore warranted, considering all female-specific predictors. Ideally their added value to models which already perform well is assessed instead of developing completely new models [12].

## Supporting information

S1 Table Articles that developed a new model in the updated search and their external validation. (DOCX)

S2 Table Models validated in the update. (DOCX)

S3 Table Models used for incremental value in the update. (DOCX)

S1 Text Full list of included papers from the update. (DOCX)

S2 Text PRISMA 2009 checklist. (DOCX)
