# Supplementary material for: Cardiovascular risk prediction models for women in the general population: A systematic review
Source: PLoS One. 2019 Jan 8;14(1):e0210329. doi: 10.1371/journal.pone.0210329 (PMC6324808; doi:10.1371/journal.pone.0210329)
Supplement: S1 Table — (DOCX) [file pone.0210329.s001.docx]

**Supplemental Table 1. Articles that developed a new model in the updated search and their external validation**

| **First author, publication year** | **Number of models developed** | **Female-specific or sex-predictor** | **Number of articles in which model is validated** |
| --- | --- | --- | --- |
| Artigao-Rodenas, 2015 | 1 | Sex-predictor | 1 (Artigao-Rodenas, 2015) |
| Backholer, 2017 | 3 | Sex-predictor | 1 (Backholer, 2017) |
| Bali, 2016 | 1 | Sex-predictor | 1 (Bali, 2016) |
| Borglykke, 2010 | 5 | Female-specific | 1 (Borglykke, 2010) |
| Chahal, 2015 | 7 | Sex-predictor | - |
| Chiuve, 2014 | 1 | Female-specific | - |
| Cooney, 2012 | 1 | Female-specific | 1 (Cooney, 2012) |
| Cross, 2013 | 1 | Sex-predictor | - |
| Deo, 2016 | 1 | Sex-predictor | 1 (Deo, 2016) |
| Dhoble, 2014 | 4 | Sex-predictor | - |
| Fox, 2016 | 6 | Sex-predictor | 1 (Fox, 2016) |
| Goff, 2013 | 2 (PCE) | Female-specific | 20 (Muntner, 2014; Lee, 2015; Khalili, 2015; Kavousi, 2014; Jung, 2015; DeFilippis, 2015; Chia, 2014; Andersson, 2015; Yang, 2016; Cook, 2014; De Las Heras Gala, 2016; DeFilippis, 2017; Emdin, 2017; Foraker, 2016; Goff, 2013; Karmali, 2015; Mortensen, 2017; Qureshi, 2016; Rana, 2016; Zhang, 2017) |
| Hajifathalian, 2015 | 2 | Female-specific | 1 (Hajifathalian, 2015) |
| Hensley, 1998 | 1 | Female-specific | - |
| Hippisley-Cox, 2013 | 1 (QSTROKE) | Female-specific | 2 (Hippisley-Cox, 2014; Parmar, 2015) |
| Hippisley-Cox, 2017 | 3 | Female-specific | 1 (Hippisley-Cox, 2017) |
| Ho, 2016 | 2 | Sex-predictor | 1 (Ho, 2016) |
| Howard, 2017 | 1 | Sex-predictor | 1 (Howard, 2017) |
| Hu, 2014 | 1 | Sex-predictor | - |
| Jairam, 2015 | 1 | Sex-predictor |  |
| Jee, 2014 | 4 | Female-specific | - |
| Johansson | 1 | Sex-predictor |  |
| Jung, 2015 | 1 | Female-specific | - |
| Kovalchik, 2013 | 1 | Female-specific | 1 (Kovalchik, 2013) |
| Kusmana, 2002 | 1 | Sex-predictor | - |
| Liu, 2016 | 1 | Female-specific |  |
| Manuel, 2015 | 1 | Female-specific |  |
| Marino, 2014 | 2 | Female-specific | - |
| Marrugat, 2014 | 6 | Female-specific | 1 (Marrugat, 2014) |
| McClelland, 2015 | 2 | Sex-predictor | 1 (McClelland, 2015) |
| McNeil, 2001 | 2 | Female-specific | - |
| Nishimura, 2014 | 4 | Sex-predictor | - |
| Nobel, 2014 | 1 | Sex-predictor | - |
| Onat, 2017 | 1 | Female-specific |  |
| Parikh, 2016 | 1 | Female-specific | - |
| Parmar, 2014 | 1 | Sex-predictor | 1 (Parmar, 2014) |
| Paynter, 2014 | 3 | Female-specific | - |
| Piotrowski, 2016 | 2 | Female-specific |  |
| Selmer, 2017 | 1 | Female-specific |  |
| Stam-Slob, 2017 | 1 | Sex-predictor |  |
| Vartiainen, 2016 | 3 | Female-specific | - |
| Wang, 2016 | 1 | Female-specific |  |
| Wickramasinghe, 2014 | 1 | Female-specific | - |
| Woodward, 2007 | 1 | Female-specific |  |
| Woodward, 2006 | 2 | Female-specific | - |
| Würtz, 2015 | 2 | Sex-predictor | 1 (Würtz, 2015) |
| Yang, 2016 | 1 | Female-specific | - |
| Yatsuya, 2016 | 2 | Sex-predictor | - |
| Yudkin, 1999 | 1 | Female-specific | - |
