# Supplementary material for: Cardiovascular risk prediction models for women in the general population: A systematic review
Source: PLoS One. 2019 Jan 8;14(1):e0210329. doi: 10.1371/journal.pone.0210329 (PMC6324808; doi:10.1371/journal.pone.0210329)
Supplement: S2 Table — (DOCX) [file pone.0210329.s002.docx]

**Supplemental Table 2. Models validated in the update**

| **Model Validated** | **Author, year model developed** | **Number of articles in which model is validated** |
| --- | --- | --- |
| Framingham | Anderson - 1991 | 2 (Goh, 2014; Tilin, 2014) |
| Framingham | ATP III - 2002 | 5 (DeFilippis, 2015; Dhoble, 2014; Hu, 2014; Qureshi, 2016; Kavousi, 2014) |
| YDR | Colditz - 2000 | 1 (De Vito, 2015) |
| SCORE | Conroy - 2003 | 15 (Goh, 2014; Jdanov, 2014; Jorstad 2014; Kavousi, 2014; Mortsensen, 2015; Selvarajah, 2014; Vikhireva, 2014-a; Vikhireva, 2014-b, Baena-Diez, 2017; Mortensen, 2017; De Las Heras Gala, 2016; Qureshi, 2016; Berard, 2016; Sawano, 2016; Piotrowski, 2016) |
| SSVMod | Counsell - 2002 | 1 (Sim, 2016) |
| Framingham | D'Agostino - 2008 | 9 (Artigao-Rodenas, 2013; Chia, 2015; DeFilipis, 2015; Marino, 2014; Selvarajah, 2014, Fatema, 2016, Qureshi, 2016; Chamnan, 2016, Sepanlou, 2015) |
| CHADS2 | Gage - 2001 | 1 (Yuan, 2017) |
| QRISK2 | Hippisley-Cox - 2008 | 2 (Hippisley-Cox, 2014; Tilin, 2014) |
| CBC Score | Horne - 2009 | 1 (Horne, 2015) |
| SCORE – Germany | Keil – 2005 | 1 (Rucker, 2016) |
| CHA2DS2 - VASC | Lip - 2010 | 1 (Yuan, 2017) |
| Framingham - Regicor | Marrugat - 2003 | 1 (Marrugat, 2014) |
| HellenicSCORE | Panagiotakos - 2007 | 1 (Panagiotakos, 2015) |
| Framingham | Pencina - 2009 | 1 (van Kempen, 2014) |
| Reynolds Risk | Ridker - 2007 | 1 (DeFilippis, 2015) |
| Dubbo | Simons - 2003 | 1 (Weatherley, 2011) |
| SCORE-NL | Van Dis - 2010 | 1 (Van Dis, 2014) |
| NIHSSMod | Weimar – 2004 | 1 (Sim, 2016) |
| WHO/ISH | WHO - 2007 | 2 (Raghu, 2015; Selvarajah, 2014) |
| SCORE – Sweden | Wilhelmsen - 2004 | 1 (Karjalainen, 2017) |
| Framingham | Wilson - 1998 | 4 (DeFilippis, 2015; Hu, 2914; Nishimura, 2014; Fowkes, 2014) |
| Framingham | Wolf - 1991 | 6 (Hippisley-Cox, 2013; McClure, 2014; Parmar, 2014; Sabayan, 2013; Dufouil, 2017; Howard, 2017) |
