# Supplementary material for: Cardiovascular risk prediction models for women in the general population: A systematic review
Source: PLoS One. 2019 Jan 8;14(1):e0210329. doi: 10.1371/journal.pone.0210329 (PMC6324808; doi:10.1371/journal.pone.0210329)
Supplement: S3 Table — (DOCX) [file pone.0210329.s003.docx]

**Supplemental Table 3. Models used for incremental value in the update.**

| **Model used for incremental value** | **Author, year model developed** | **Number of articles used for IV** |
| --- | --- | --- |
| ARIC HF | Agarwal - 2012 | 1 (Nambi, 2013) |
| Framingham | Anderson - 1991 | 1 (Wassertheil-Smel, 2014) |
| Framingham | ATP III - 2002 | 2 (Hadamitzky, 2013; Valentini, 2015) |
| SCORE | Conroy - 2003 | 7 (Faeh, 2013; Ferrario, 2014; Groot, 2015; Schnohr, 2015; Sehestedt, 2011; Vikhireva, 2014; Woznicka-Leskiew, 2015) |
| Framingham | Cupples - 1988 | 1 (Lluis-Ganella, 2012) |
| Framingham | D'Agostino - 1994 | 2 (Gibson, 2014; Ziegelbauer) |
| Framingham | D'Agostino - 2000 | 1 (Aljaroudi, 2013) |
| Framingham | D'Agostino - 2001 | 1 (Yeboah, 2014) |
| Framingham | D'Agostino - 2008 | 5 (Armstrong, 2014; Criqui, 2013; Goh, 2014; Kunutsor, 2015; Lopez-Suarez, 2014) |
| AGLA | Eckardstein - 2012 | 1 (Romanens, 2014) |
| -- | Ferrario -2005 | 1 (Veronesi, 2014) |
| Framingham | Unspecified | 8 (Badheka, 2013-a; Badheka, 2013-b; Brouwers, 2014; Gaibazzi, 2014; Lindberg, 2014; Okwuosa, 2014; Willeit, 2014; Woznicka-Leskiew, 2015) |
| Pooled Cohort Equations | Goff - 2013 | 3 (Everett, 2015; Kim, 2014; Okwuosa, 2014) |
| QRISK2 | Hippisley-Cox - 2008 | 1 (Weng, 2015) |
| REGICOR | Marrugat - 2003 | 2 (Velescu, 2015; Llius-Ganella, 2012) |
| Laboratory Report Model | Nambi - 2013 | Nambi - 2013 |
| HellenicSCORE | Panagiotakos - 2007 | 1 (Georgousopoulou, 2015) |
| Reynolds Risk | Ridker - 20017 | 4 (Everett, 2015; Everett, 2014; Kim, 2014; Shah, 2014) |
| NL-SCORE | Smulders - 2008 | 1 (Van Dis, 2012) |
| Traditional Risk Factors | - | 7 (Baber, 2015; Candell-Riera, 2013; Funke-Kaiser, 2014; Gardin, 2014; Kunutsor, 2014; Nielson, 2014; Nimomiya, 2013) |
| Framingham | Wilson - 1998 | 11 (Bérard, 2013; Britton, 2013; Fowkes, 2014; Gronewold, 2014; Kalsch, 2014; Lyngbaek, 2012; Mahabadi, 2015; Polak, 2015; Valentini, 2015; Weng, 2015; Zalawadiya, 2015) |
