# Supplementary material for: Cardiovascular risk prediction models for women in the general population: A systematic review
Source: PLoS One. 2019 Jan 8;14(1):e0210329. doi: 10.1371/journal.pone.0210329 (PMC6324808; doi:10.1371/journal.pone.0210329)
Supplement: S1 Text — (DOCX) [file pone.0210329.s004.docx]

**Supplemental Text 1. Full list of included papers from the update.**

1. Abbasi A, Kieneker LM, Corpeleijn E, Gansevoort RT, Gans RO, Struck J, de Boer RA, Hillege HL, Stolk RP, Navis G and Bakker SJ. Plasma N-terminal Prosomatostatin and Risk of Incident Cardiovascular Disease and All-Cause Mortality in a Prospective Observational Cohort: the PREVEND Study. *Clin Chem*. 2017;63:278-287.

2. Abraham G, Havulinna AS, Bhalala OG, Byars SG, De Livera AM, Yetukuri L, Tikkanen E, Perola M, Schunkert H, Sijbrands EJ, Palotie A, Samani NJ, Salomaa V, Ripatti S and Inouye M. Genomic prediction of coronary heart disease. *Eur Heart J*. 2016;37:3267-3278.

3. Aijala M, Ronkainen J, Huusko T, Malo E, Savolainen ER, Savolainen MJ, Salonurmi T, Bloigu R, Kesaniemi YA and Ukkola O. The fat mass and obesity-associated (FTO) gene variant rs9939609 predicts long-term incidence of cardiovascular disease and related death independent of the traditional risk factors. *Ann Med*. 2015;47:655-63.

4. AlJaroudi WA, Alraies MC, Halley C, Menon V, Rodriguez LL, Grimm RA, Thomas JD and Jaber WA. Incremental prognostic value of diastolic dysfunction in low risk patients undergoing echocardiography: beyond Framingham score. *Int J Cardiovas Imag*. 2013;29:1441-1450.

5. Amato M, Veglia F, de Faire U, Giral P, Rauramaa R, Smit AJ, Kurl S, Ravani A, Frigerio B, Sansaro D, Bonomi A, Tedesco CC, Castelnuovo S, Mannarino E, Humphries SE, Hamsten A, Tremoli E, Baldassarre D and group Is. Carotid plaque-thickness and common carotid IMT show additive value in cardiovascular risk prediction and reclassification. *Atherosclerosis*. 2017;263:412-419.

6. Ambale-Venkatesh B, Yoneyama K, Sharma RK, Ohyama Y, Wu CO, Burke GL, Shea S, Gomes AS, Young AA, Bluemke DA and Lima JAC. Left ventricular shape predicts different types of cardiovascular events in the general population. *Heart*. 2017;103:507-515.

7. Andersson C, Enserro D, Larson MG, Xanthakis V and Vasan RS. Implications of the US cholesterol guidelines on eligibility for statin therapy in the community: comparison of observed and predicted risks in the Framingham Heart Study Offspring Cohort. *Journal of the American Heart Association*. 2015;4(4).

8. Armstrong AC, Liu K, Lewis CE, Sidney S, Colangelo LA, Kishi S, Ambale-Venkatesh B, Arynchyn A, Jacobs DR, Jr., Correia LC, Gidding SS and Lima JA. Left atrial dimension and traditional cardiovascular risk factors predict 20-year clinical cardiovascular events in young healthy adults: the CARDIA study. *Eur Heart J Cardiovasc Imaging*. 2014;15:893-9.

9. Artigao-Rodenas LM, Carbayo-Herencia JA, Divison-Garrote JA, Gil-Guillen VF, Masso-Orozco J, Simarro-Rueda M, Molina-Escribano F, Sanchis C, Carrion-Valero L, Lopez de Coca E, Caldevilla D, Lopez-Abril J, Carratala-Munuera C, Lopez-Pineda A and Grupo de Enfermedades Vasculares de A. Framingham risk score for prediction of cardiovascular diseases: a population-based study from southern Europe. *PLoS ONE*. 2013;8:e73529.

10. Artigao-Rodenas LM, Carbayo-Herencia JA, Palazon-Bru A, Divison-Garrote JA, Sanchis-Domenech C, Vigo-Aguiar I and Gil-Guillen VF. Construction and Validation of a 14-Year Cardiovascular Risk Score for Use in the General Population: The Puras-GEVA Chart. *Medicine*. 2015;94:e1980.

11. Astor BC, Shafi T, Hoogeveen RC, Matsushita K, Ballantyne CM, Inker LA and Coresh J. Novel Markers of Kidney Function as Predictors of ESRD, Cardiovascular Disease, and Mortality in the General Population. *Am J Kidney Dis*. 2012;59:653-662.

12. Baber U, Mehran R, Sartori S, Schoos MM, Sillesen H, Muntendam P, Garcia MJ, Gregson J, Pocock S, Falk E and Fuster V. Prevalence, impact, and predictive value of detecting subclinical coronary and carotid atherosclerosis in asymptomatic adults: the BioImage study. *J Am Coll Cardiol*. 2015;65:1065-74.

13. Backholer K, Hirakawa Y, Tonkin A, Giles G, Magliano DJ, Colagiuri S, Harris M, Mitchell P, Nelson M, Shaw JE, Simmons D, Simons L, Taylor A, Harding J, Gopinath B and Woodward M. Development of an Australian cardiovascular disease mortality risk score using multiple imputation and recalibration from national statistics. *BMC Cardiovascular Disorders*. 2017;17:17.

14. Badheka AO, Patel NJ, Grover PM, Shah N, Singh V, Deshmukh A, Mehta K, Chothani A, Hoosien M, Rathod A, Savani GT, Marzouka GR, Gupta S, Mitrani RD, Moscucci M and Cohen MG. ST-T wave abnormality in lead aVR and reclassification of cardiovascular risk (from the National Health and Nutrition Examination Survey-III). *American Journal of Cardiology*. 2013;112:805-10.

15. Badheka AO, Singh V, Patel NJ, Deshmukh A, Shah N, Chothani A, Mehta K, Grover P, Savani GT, Gupta S, Rathod A, Marzouka GR, Mitrani RD, Moscucci M and Cohen MG. QRS duration on electrocardiography and cardiovascular mortality (from the National Health and Nutrition Examination Survey-III). *American Journal of Cardiology*. 2013;112:671-7.

16. Baena-Diez JM, Subirana I, Ramos R, Gomez de la Camara A, Elosua R, Vila J, Marin-Ibanez A, Guembe MJ, Rigo F, Tormo-Diaz MJ, Moreno-Iribas C, Cabre JJ, Segura A, Lapetra J, Quesada M, Medrano MJ, Gonzalez-Diego P, Frontera G, Gavrila D, Ardanaz E, Basora J, Garcia JM, Garcia-Lareo M, Gutierrez-Fuentes JA, Mayoral E, Sala J, I RD, Frances A, Castell C, Grau M and Marrugat J. Validity Assessment of Low-risk SCORE Function and SCORE Function Calibrated to the Spanish Population in the FRESCO Cohorts. *Rev Esp Cardiol (Engl Ed)*. 2017.

17. Bali V, Yermilov I, Coutts K and Legorreta AP. Novel screening metric for the identification of at-risk peripheral artery disease patients using administrative claims data. *Vascular Medicine*. 2016;21:33-40.

18. Barr EL, Reutens A, Magliano DJ, Wolfe R, Lu ZX, Sikaris KA, Tanamas SK, Atkins R, Chadban S, Shaw JE and Polkinghorne KR. Cystatin C estimated glomerular filtration rate and all-cause and cardiovascular disease mortality risk in the general population: AusDiab study. *Nephrology (Carlton)*. 2017;22:243-250.

19. Bellinazzi VR, Cipolli JA, Pimenta MV, Guimaraes PV, Pio-Magalhaes JA, Coelho-Filho OR, Biering-Sorensen T, Matos-Souza JR, Sposito AC and Nadruz W, Jr. Carotid flow velocity/diameter ratio is a predictor of cardiovascular events in hypertensive patients. *J Hypertens*. 2015;33:2054-60.

20. Berard E, Bongard V, Ruidavets JB, Amar J and Ferrieres J. Pulse wave velocity, pulse pressure and number of carotid or femoral plaques improve prediction of cardiovascular death in a population at low risk. *J Hum Hypertens*. 2013;27:529-534.

21. Berard E, Seguro F, Bongard V, Dallongeville J, Arveiler D, Amouyel P, Wagner A, Ruidavets JB and Ferrieres J. Predictive Accuracy of the European Society of Cardiology SCORE Among French People. *Journal of Cardiopulmonary Rehabilitation & Prevention*. 2016;36:38-48.

22. Biering-Sorensen T, Biering-Sorensen SR, Olsen FJ, Sengelov M, Jorgensen PG, Mogelvang R, Shah AM and Jensen JS. Global Longitudinal Strain by Echocardiography Predicts Long-Term Risk of Cardiovascular Morbidity and Mortality in a Low-Risk General Population: The Copenhagen City Heart Study. *Circ Cardiovasc Imaging*. 2017;10.

23. Biering-Sorensen T, Mogelvang R, Schnohr P and Jensen JS. Cardiac Time Intervals Measured by Tissue Doppler Imaging M-mode: Association With Hypertension, Left Ventricular Geometry, and Future Ischemic Cardiovascular Diseases. *Journal of the American Heart Association*. 2016;5.

24. Blaha MJ, Budoff MJ, Tota-Maharaj R, Dardari ZA, Wong ND, Kronmal RA, Eng J, Post WS, Blumenthal RS and Nasir K. Improving the CAC Score by Addition of Regional Measures of Calcium Distribution: Multi-Ethnic Study of Atherosclerosis. *JACC Cardiovasc Imaging*. 2016;9:1407-1416.

25. Blankenberg S, Salomaa V, Makarova N, Ojeda F, Wild P, Lackner KJ, Jorgensen T, Thorand B, Peters A, Nauck M, Petersmann A, Vartiainen E, Veronesi G, Brambilla P, Costanzo S, Iacoviello L, Linden G, Yarnell J, Patterson CC, Everett BM, Ridker PM, Kontto J, Schnabel RB, Koenig W, Kee F, Zeller T, Kuulasmaa K and Investigators B. Troponin I and cardiovascular risk prediction in the general population: the BiomarCaRE consortium. *Eur Heart J*. 2016;37:2428-+.

26. Borglykke A, Andreasen AH, Kuulasmaa K, Sans S, Ducimetiere P, Vanuzzo D, Ferrario MM, Palmieri L, Karvanen J, Tunstall-Pedoe H, Jorgensen T and Project M. Stroke risk estimation across nine European countries in the MORGAM project. *Heart*. 2010;96:1997-2004.

27. Bos D, Ikram MA, Leening MJG and Ikram MK. The Revised Framingham Stroke Risk Profile in a Primary Prevention Population: The Rotterdam Study. *Circulation*. 2017;135:2207-2209.

28. Bozorgmanesh M, Sardarinia M, Hajsheikholeslami F, Azizi F and Hadaegh F. CVD-predictive performances of "a body shape index" versus simple anthropometric measures: Tehran lipid and glucose study. *Eur J Nutr*. 2016;55:147-157.

29. Britton KA, Massaro JM, Murabito JM, Kreger BE, Hoffmann U and Fox CS. Body fat distribution, incident cardiovascular disease, cancer, and all-cause mortality. *J Am Coll Cardiol*. 2013;62:921-5.

30. Brouwers FP, Asselbergs FW, Hillege HL, Gansevoort RT, de Boer RA and van Gilst WH. Elevated urinary albumin excretion complements the Framingham Risk Score for the prediction of cardiovascular risk - response to treatment in the PREVEND IT trial. *IJC Heart and Vessels*. 2014;4:193-197.

31. Brownrigg JR, Hughes CO, Burleigh D, Karthikesalingam A, Patterson BO, Holt PJ, Thompson MM, de Lusignan S, Ray KK and Hinchliffe RJ. Microvascular disease and risk of cardiovascular events among individuals with type 2 diabetes: a population-level cohort study. *Lancet Diabetes Endocrinol*. 2016;4:588-97.

32. Brutsaert EF, Shitole S, Biggs ML, Mukamal KJ, deBoer IH, Thacker EL, Barzilay JI, Djousse L, Ix JH, Smith NL, Kaplan RC, Siscovick DS, Psaty BM and Kizer JR. Relations of Postload and Fasting Glucose With Incident Cardiovascular Disease and Mortality Late in Life: The Cardiovascular Health Study. *J Gerontol A Biol Sci Med Sci*. 2016;71:370-7.

33. Bye A, Rosjo H, Nauman J, Silva GJ, Follestad T, Omland T and Wisloff U. Circulating microRNAs predict future fatal myocardial infarction in healthy individuals - The HUNT study. *J Mol Cell Cardiol*. 2016;97:162-8.

34. Candell-Riera J, Ferreira-Gonzalez I, Marsal JR, Aguade-Bruix S, Cuberas-Borros G, Pujol P, Romero-Farina G, Nazarena-Pizzi M, de Leon G, Castell-Conesa J and Garcia-Dorado D. Usefulness of exercise test and myocardial perfusion-gated single photon emission computed tomography to improve the prediction of major events. *Circ Cardiovasc Imaging*. 2013;6:531-41.

35. Chahal H, Bluemke DA, Wu CO, McClelland R, Liu K, Shea SJ, Burke G, Balfour P, Herrington D, Shi P, Post W, Olson J, Watson KE, Folsom AR and Lima JA. Heart failure risk prediction in the Multi-Ethnic Study of Atherosclerosis. *Heart*. 2015;101:58-64.

36. Chambless LE, Folsom AR, Sharrett AR, Sorlie P, Couper D, Szklo M and Nieto FJ. Coronary heart disease risk prediction in the Atherosclerosis Risk in Communities (ARIC) study. *J Clin Epidemiol*. 2003;56:880-90.

37. Chamnan P, Simmons RK, Sharp SJ, Khaw KT, Wareham NJ and Griffin SJ. Repeat Cardiovascular Risk Assessment after Four Years: Is There Improvement in Risk Prediction? *PLoS One*. 2016;11:e0147417.

38. Chang X, Salim A, Dorajoo R, Han Y, Khor CC, van Dam RM, Yuan JM, Koh WP, Liu J, Goh DY, Wang X, Teo YY, Friedlander Y and Heng CK. Utility of genetic and non-genetic risk factors in predicting coronary heart disease in Singaporean Chinese. *Eur J Prev Cardiol*. 2017;24:153-160.

39. Chia YC, Gray SYW, Ching SM, Lim HM and Chinna K. Validation of the Framingham general cardiovascular risk score in a multiethnic Asian population: a retrospective cohort study. *Bmj Open*. 2015;5.

40. Chia YC, Lim HM and Ching SM. Validation of the pooled cohort risk score in an Asian population - a retrospective cohort study. *BMC Cardiovascular Disorders*. 2014;14.

41. Chiuve SE, Cook NR, Shay CM, Rexrode KM, Albert CM, Manson JE, Willett WC and Rimm EB. Lifestyle-based prediction model for the prevention of CVD: the Healthy Heart Score. *Journal of the American Heart Association*. 2014;3:e000954.

42. Cho I, Chang HJ, B OH, Shin S, Sung JM, Lin FY, Achenbach S, Heo R, Berman DS, Budoff MJ, Callister TQ, Al-Mallah MH, Cademartiri F, Chinnaiyan K, Chow BJ, Dunning AM, DeLago A, Villines TC, Hadamitzky M, Hausleiter J, Leipsic J, Shaw LJ, Kaufmann PA, Cury RC, Feuchtner G, Kim YJ, Maffei E, Raff G, Pontone G, Andreini D and Min JK. Incremental prognostic utility of coronary CT angiography for asymptomatic patients based upon extent and severity of coronary artery calcium: results from the COronary CT Angiography EvaluatioN For Clinical Outcomes InteRnational Multicenter (CONFIRM) study. *Eur Heart J*. 2015;36:501-8.

43. Christophersen IE, Yin X, Larson MG, Lubitz SA, Magnani JW, McManus DD, Ellinor PT and Benjamin EJ. A comparison of the CHARGE-AF and the CHA2DS2-VASc risk scores for prediction of atrial fibrillation in the Framingham Heart Study. *American Heart Journal*. 2016;178:45-54.

44. Colantonio LD, Richman JS, Carson AP, Lloyd-Jones DM, Howard G, Deng L, Howard VJ, Safford MM, Muntner P and Goff DC, Jr. Performance of the Atherosclerotic Cardiovascular Disease Pooled Cohort Risk Equations by Social Deprivation Status. *Journal of the American Heart Association*. 2017;6.

45. Cook NR and Ridker PM. Further Insight Into the Cardiovascular Risk Calculator The Roles of Statins, Revascularizations, and Underascertainment in the Women's Health Study. *Jama Intern Med*. 2014;174:1964-1971.

46. Cooney MT, Vartiainen E, Laatikainen T, De Bacquer D, McGorrian C, Dudina A, Graham I, Score and investigators F. Cardiovascular risk age: concepts and practicalities. *Heart*. 2012;98:941-6.

47. Criqui MH, Denenberg JO, Ix JH, McClelland RL, Wassel CL, Rifkin DE, Carr JJ, Budoff MJ and Allison MA. Calcium density of coronary artery plaque and risk of incident cardiovascular events. *JAMA*. 2014;311:271-8.

48. Cross DS, McCarty CA, Steinhubl SR, Carey DJ and Erlich PM. Development of a multi-institutional cohort to facilitate cardiovascular disease biomarker validation using existing biorepository samples linked to electronic health records. *Clinical Cardiology*. 2013;36:486-91.

49. Daniels LB, Clopton P, deFilippi CR, Sanchez OA, Bahrami H, Lima JA, Tracy RP, Siscovick D, Bertoni AG, Greenland P, Cushman M, Maisel AS and Criqui MH. Serial measurement of N-terminal pro-B-type natriuretic peptide and cardiac troponin T for cardiovascular disease risk assessment in the Multi-Ethnic Study of Atherosclerosis (MESA). *American Heart Journal*. 2015;170:1170-1183.

50. De Las Heras Gala T, Geisel MH, Peters A, Thorand B, Baumert J, Lehmann N, Jockel KH, Moebus S, Erbel R, Mahabadi AA, Koenig W, Heinrich J, Holle R, Leidl R, Meisinger C, Strauch K, Roggenbuck U, Slomiany U, Beck EM, Offner A, Munkel S, Bauer M, Schrader S, Peter R and Hirche H. Recalibration of the ACC/AHA risk score in two population-based German cohorts. *PLoS ONE*. 2016;11:e0164688.

51. de Lemos JA, Ayers CR, Levine B, deFilippi CR, Wang TJ, Hundley WG, Berry JD, Seliger SL, McGuire DK, Ouyang P, Drazner MH, Budoff M, Greenland P, Ballantyne CM and Khera A. Multimodality Strategy for Cardiovascular Risk Assessment: Performance in 2 Population-Based Cohorts. *Circulation*. 2017;135:2119-2132.

52. De Vito KM, Baer HJ, Dart H, Chiuve SE, Rimm EB and Colditz GA. Validation of a risk prediction tool for coronary heart disease in middle-aged women. *BMC Womens Health*. 2015;15:101.

53. DeFilippis AP, Young R and Blaha MJ. Calibration and Discrimination Among Multiple Cardiovascular Risk Scores in a Modern Multiethnic Cohort RESPONSE. *Ann Intern Med*. 2015;163:68-69.

54. DeFilippis AP, Young R, McEvoy JW, Michos ED, Sandfort V, Kronmal RA, McClelland RL and Blaha MJ. Risk score overestimation: the impact of individual cardiovascular risk factors and preventive therapies on the performance of the American Heart Association-American College of Cardiology-Atherosclerotic Cardiovascular Disease risk score in a modern multi-ethnic cohort. *Eur Heart J*. 2017;38:598-608.

55. Deo R, Norby FL, Katz R, Sotoodehnia N, Adabag S, DeFilippi CR, Kestenbaum B, Chen LY, Heckbert SR, Folsom AR, Kronmal RA, Konety S, Patton KK, Siscovick D, Shlipak MG and Alonso A. Development and Validation of a Sudden Cardiac Death Prediction Model for the General Population. *Circulation*. 2016;134:806-16.

56. Desai CS, Bartz TM, Gottdiener JS, Lloyd-Jones DM and Gardin JM. Usefulness of Left Ventricular Mass and Geometry for Determining 10-Year Prediction of Cardiovascular Disease in Adults Aged >65 Years (from the Cardiovascular Health Study). *American Journal of Cardiology*. 2016;118:684-90.

57. Dhoble A, Lahr BD, Allison TG, Bailey KR, Thomas RJ, Lopez-Jimenez F, Kullo IJ, Gupta B and Kopecky SL. Predicting long-term cardiovascular risk using the mayo clinic cardiovascular risk score in a referral population. *American Journal of Cardiology*. 2014;114:704-10.

58. Di Angelantonio E, Gao P, Khan H, Butterworth AS, Wormser D, Kaptoge S, Kondapally Seshasai SR, Thompson A, Sarwar N, Willeit P, Ridker PM, Barr ELM, Khaw KT, Psaty BM, Brenner H, Balkau B, Dekker JM, Lawlor DA, Daimon M, Willeit J, Njolstad I, Nissinen A, Brunner EJ, Kuller LH, Price JF, Sundstrom J, Knuiman MW, Feskens EJM, Verschuren WMM, Wald N, Bakker SJL, Whincup PH, Ford I, Goldbourt U, Gomez-de-la-Camara A, Gallacher J, Simons LA, Rosengren A, Sutherland SE, Bjorkelund C, Blazer DG, Wassertheil-Smoller S, Onat A, Marin Ibanez A, Casiglia E, Jukema JW, Simpson LM, Giampaoli S, Nordestgaard BG, Selmer R, Wennberg P, Kauhanen J, Salonen JT, Dankner R, Barrett-Connor E, Kavousi M, Gudnason V, Evans D, Wallace RB, Cushman M, D'Agostino RB, Umans JG, Kiyohara Y, Nakagawa H, Sato S, Gillum RF, Folsom AR, van der Schouw YT, Moons KG, Griffin SJ, Sattar N, Wareham NJ, Selvin E, Thompson SG and Danesh J. Glycated hemoglobin measurement and prediction of cardiovascular disease. *JAMA - Journal of the American Medical Association*. 2014;311:1225-1233.

59. Dufouil C, Beiser A, McLure LA, Wolf PA, Tzourio C, Howard VJ, Westwood AJ, Himali JJ, Sullivan L, Aparicio HJ, Kelly-Hayes M, Ritchie K, Kase CS, Pikula A, Romero JR, D'Agostino RB, Samieri C, Vasan RS, Chene G, Howard G and Seshadri S. Revised Framingham Stroke Risk Profile to Reflect Temporal Trends. *Circulation*. 2017;135:1145-1159.

60. Eggers KM, Kempf T, Larsson A, Lindahl B, Venge P, Wallentin L, Wollert KC and Lind L. Evaluation of Temporal Changes in Cardiovascular Biomarker Concentrations Improves Risk Prediction in an Elderly Population from the Community. *Clin Chem*. 2016;62:485-93.

61. Emdin CA, Khera AV, Natarajan P, Klarin D, Baber U, Mehran R, Rader DJ, Fuster V and Kathiresan S. Evaluation of the Pooled Cohort Equations for Prediction of Cardiovascular Risk in a Contemporary Prospective Cohort. *American Journal of Cardiology*. 2017;119:881-885.

62. Evans TE, O'Sullivan MJ, de Groot M, Niessen WJ, Hofman A, Krestin GP, van der Lugt A, Portegies ML, Koudstaal PJ, Bos D, Vernooij MW and Ikram MA. White Matter Microstructure Improves Stroke Risk Prediction in the General Population. *Stroke*. 2016;47:2756-2762.

63. Everett BM, Berger JS, Manson JE, Ridker PM and Cook NR. B-type natriuretic peptides improve cardiovascular disease risk prediction in a cohort of women. *J Am Coll Cardiol*. 2014;64:1789-97.

64. Everett BM, Ridker PM, Cook NR and Pradhan AD. Usefulness of B-type Natriuretic Peptides to Predict Cardiovascular Events in Women (from the Women's Health Study). *American Journal of Cardiology*. 2015;116:532-7.

65. Faeh D, Rohrmann S and Braun J. Better risk assessment with glycated hemoglobin instead of cholesterol in CVD risk prediction charts. *Eur J Epidemiol*. 2013;28:551-5.

66. Fatema K, Rahman B, Zwar NA, Milton AH and Ali L. Short-term predictive ability of selected cardiovascular risk prediction models in a rural Bangladeshi population: a case-cohort study. *BMC Cardiovascular Disorders*. 2016;16:105.

67. Ferrario MM, Veronesi G, Chambless LE, Tunstall-Pedoe H, Kuulasmaa K, Salomaa V, Borglykke A, Hart N, Soderberg S, Cesana G and Project M. The contribution of educational class in improving accuracy of cardiovascular risk prediction across European regions: The MORGAM Project Cohort Component. *Heart*. 2014;100:1179-87.

68. Fogacci F, Cicero AF, D'Addato S, D'Agostini L, Rosticci M, Giovannini M, Bertagnin E, Borghi C and Brisighella Heart Study G. Serum lipoprotein(a) level as long-term predictor of cardiovascular mortality in a large sample of subjects in primary cardiovascular prevention: data from the Brisighella Heart Study. *Eur J Intern Med*. 2017;37:49-55.

69. Foraker RE, Greiner M, Sims M, Tucker KL, Towfighi A, Bidulescu A, Shoben AB, Smith S, Talegawkar S, Blackshear C, Wang W, Hardy NC and O'Brien E. Comparison of risk scores for the prediction of stroke in African Americans: Findings from the Jackson Heart Study. *Am Heart J*. 2016;177:25-32.

70. Fowkes FG, Murray GD, Butcher I, Folsom AR, Hirsch AT, Couper DJ, Debacker G, Kornitzer M, Newman AB, Sutton-Tyrrell KC, Cushman M, Lee AJ, Price JF, D'Agostino RB, Sr., Murabito JM, Norman P, Masaki KH, Bouter LM, Heine RJ, Stehouwer CD, McDermott MM, Stoffers HE, Knottnerus JA, Ogren M, Hedblad B, Koenig W, Meisinger C, Cauley JA, Franco O, Hunink MG, Hofman A, Witteman JC, Criqui MH, Langer RD, Hiatt WR, Hamman RF and Ankle Brachial Index C. Development and validation of an ankle brachial index risk model for the prediction of cardiovascular events. *Eur J Prev Cardiolog*. 2014;21:310-20.

71. Fox ER, Samdarshi TE, Musani SK, Pencina MJ, Sung JH, Bertoni AG, Xanthakis V, Balfour PC, Jr., Shreenivas SS, Covington C, Liebson PR, Sarpong DF, Butler KR, Mosley TH, Rosamond WD, Folsom AR, Herrington DM, Vasan RS and Taylor HA. Development and Validation of Risk Prediction Models for Cardiovascular Events in Black Adults: The Jackson Heart Study Cohort. *JAMA Cardiol*. 2016;1:15-25.

72. Funke-Kaiser A, Havulinna AS, Zeller T, Appelbaum S, Jousilahti P, Vartiainen E, Blankenberg S, Sydow K and Salomaa V. Predictive value of midregional pro-adrenomedullin compared to natriuretic peptides for incident cardiovascular disease and heart failure in the population-based FINRISK 1997 cohort. *Annals of Medicine*. 2014;46:155-162.

73. Gaibazzi N, Rigo F, Facchetti R, Carerj S, Giannattasio C, Moreo A, Mureddu G, Paini A, Grolla E, Faden G, Cesana F and Faggiano P. Ultrasound carotid intima-media thickness, carotid plaque and cardiac calcium incrementally add to the Framingham Risk Score for the prediction of angiographic coronary artery disease: A multicenter prospective study. *International Journal of Cardiology*. 2014;177:708-710.

74. Garcia-Gil M, Parramon D, Comas-Cufi M, Marti R, Ponjoan A, Alves-Cabratosa L, Blanch J, Petersen I, Elosua R, Grau M, Salvador B and Ramos R. Role of renal function in cardiovascular risk assessment: A retrospective cohort study in a population with low incidence of coronary heart disease. *Prev Med*. 2016;89:200-6.

75. Gardin JM, Bartz TM, Polak JF, O'Leary DH and Wong ND. What do carotid intima-media thickness and plaque add to the prediction of stroke and cardiovascular disease risk in older adults? The cardiovascular health study. *J Am Soc Echocardiogr*. 2014;27:998-1005 e2.

76. Georgousopoulou EN, Panagiotakos DB, Bougatsas D, Chatzigeorgiou M, Kavouras SA, Chrysohoou C, Skoumas I, Tousoulis D, Stefanadis C and Pitsavos C. Physical Activity Level Improves the Predictive Accuracy of Cardiovascular Disease Risk Score: The ATTICA Study (2002-2012). *Int J Prev Med*. 2016;7:52.

77. Georgousopoulou EN, Panagiotakos DB, Pitsavos C, Stefanadis C and group As. Assessment of diet quality improves the classification ability of cardiovascular risk score in predicting future events: The 10-year follow-up of the ATTICA study (2002-2012). *Eur J Prev Cardiol*. 2015;22:1488-98.

78. Gibson AO, Blaha MJ, Arnan MK, Sacco RL, Szklo M, Herrington DM and Yeboah J. Coronary artery calcium and incident cerebrovascular events in an asymptomatic cohort. The MESA Study. *JACC Cardiovasc Imaging*. 2014;7:1108-15.

79. Goff DC, Jr., Lloyd-Jones DM, Bennett G, Coady S, D'Agostino RB, Sr., Gibbons R, Greenland P, Lackland DT, Levy D, O'Donnell CJ, Robinson JG, Schwartz JS, Shero ST, Smith SC, Jr., Sorlie P, Stone NJ, Wilson PW and American College of Cardiology/American Heart Association Task Force on Practice G. 2013 ACC/AHA guideline on the assessment of cardiovascular risk: a report of the American College of Cardiology/American Heart Association Task Force on Practice Guidelines. *J Am Coll Cardiol*. 2014;63:2935-59.

80. Goh LG, Dhaliwal SS, Welborn TA, Lee AH and Della PR. Anthropometric measurements of general and central obesity and the prediction of cardiovascular disease risk in women: a cross-sectional study. *BMJ Open*. 2014;4:e004138.

81. Goh LG, Welborn TA and Dhaliwal SS. Independent external validation of cardiovascular disease mortality in women utilising Framingham and SCORE risk models: a mortality follow-up study. *BMC Womens Health*. 2014;14:118.

82. Grau M, Subirana I, Vila J, Elosua R, Ramos R, Sala J, Degano IR, Tresserras R, Bielsa O and Marrugat J. Validation of a population coronary disease predictive system: the CASSANDRA model. *J Epidemiol Community Health*. 2014;68:1012-9.

83. Graversen P, Abildstrom SZ, Jespersen L, Borglykke A and Prescott E. Cardiovascular risk prediction: Can Systematic Coronary Risk Evaluation (SCORE) be improved by adding simple risk markers? Results from the Copenhagen City Heart Study. *Eur J Prev Cardiol*. 2016;23:1546-56.

84. Greve SV, Blicher MK, Kruger R, Sehestedt T, Gram-Kampmann E, Rasmussen S, Vishram JKK, Boutouyrie P, Laurent S and Olsen MH. Estimated carotid-femoral pulse wave velocity has similar predictive value as measured carotid-femoral pulse wave velocity. *J Hypertens*. 2016;34:1279-1289.

85. Gronewold J, Hermann DM, Lehmann N, Kroger K, Lauterbach K, Berger K, Weimar C, Kalsch HI, Moebus S, Jockel KH, Bauer M, Erbel R and Heinz Nixdorf Recall Study Investigative G. Ankle-brachial index predicts stroke in the general population in addition to classical risk factors. *Atherosclerosis*. 2014;233:545-50.

86. Groot A, Bots ML, Rutten FH, den Ruijter HM, Numans ME and Vaartjes I. Measurement of ECG abnormalities and cardiovascular risk classification: a cohort study of primary care patients in the Netherlands. *Br J Gen Pract*. 2015;65:e1-8.

87. Guarrera S, Fiorito G, Onland-Moret NC, Russo A, Agnoli C, Allione A, Di Gaetano C, Mattiello A, Ricceri F, Chiodini P, Polidoro S, Frasca G, Verschuren MWM, Boer JMA, Iacoviello L, van der Schouw YT, Tumino R, Vineis P, Krogh V, Panico S, Sacerdote C and Matullo G. Gene-specific DNA methylation profiles and LINE-1 hypomethylation are associated with myocardial infarction risk. *Clin Epigenetics*. 2015;7:133.

88. Gullu O, Tekindal MA, Ates C, Ekici B and Yavuz Y. Assessing the classification performance of the mean platelet volume (MPV) in a cardiovascular risk prediction model. *Biomedical Research (India)*. 2017;28:705-710.

89. Hadamitzky M, Achenbach S, Al-Mallah M, Berman D, Budoff M, Cademartiri F, Callister T, Chang HJ, Cheng V, Chinnaiyan K, Chow BJW, Cury R, Delago A, Dunning A, Feuchtner G, Gomez M, Kaufmann P, Kim YJ, Leipsic J, Lin FY, Maffei E, Min JK, Raff G, Shaw LJ, Villines TC, Hausleiter J and Investigators C. Optimized Prognostic Score for Coronary Computed Tomographic Angiography Results From the CONFIRM Registry (COronary CT Angiography EvaluatioN For Clinical Outcomes: An InteRnational Multicenter Registry). *Journal of the American College of Cardiology*. 2013;62:468-476.

90. Hajifathalian K, Ueda P, Lu Y, Woodward M, Ahmadvand A, Aguilar-Salinas CA, Azizi F, Cifkova R, Di Cesare M, Eriksen L, Farzadfar F, Ikeda N, Khalili D, Khang YH, Lanska V, Leon-Munoz L, Magliano D, Msyamboza KP, Oh K, Rodriguez-Artalejo F, Rojas-Martinez R, Shaw JE, Stevens GA, Tolstrup J, Zhou B, Salomon JA, Ezzati M and Danaei G. A novel risk score to predict cardiovascular disease risk in national populations (Globorisk): a pooled analysis of prospective cohorts and health examination surveys. *Lancet Diabetes Endocrinol*. 2015;3:339-55.

91. Hartaigh BO, Valenti V, Cho I, Schulman-Marcus J, Gransar H, Knapper J, Kelkar AA, Xie JX, Chang HJ, Shaw LJ, Callister TQ and Min JK. 15-Year prognostic utility of coronary artery calcium scoring for all-cause mortality in the elderly. *Atherosclerosis*. 2016;246:361-6.

92. Hensley WJ and Mansfield CH. Lipoproteins, atherogenicity, age and risk of myocardial infarction. *Australian and New Zealand Journal of Public Health*. 1999;23:174-178.

93. Hippisley-Cox J, Coupland C and Brindle P. Derivation and validation of QStroke score for predicting risk of ischaemic stroke in primary care and comparison with other risk scores: a prospective open cohort study. *BMJ*. 2013;346:f2573.

94. Hippisley-Cox J, Coupland C and Brindle P. The performance of seven QPrediction risk scores in an independent external sample of patients from general practice: a validation study. *BMJ Open*. 2014;4:e005809.

95. Hippisley-Cox J, Coupland C and Brindle P. Development and validation of QRISK3 risk prediction algorithms to estimate future risk of cardiovascular disease: prospective cohort study. *BMJ*. 2017;357:j2099.

96. Ho JE, Enserro D, Brouwers FP, Kizer JR, Shah SJ, Psaty BM, Bartz TM, Santhanakrishnan R, Lee DS, Chan C, Liu K, Blaha MJ, Hillege HL, van der Harst P, van Gilst WH, Kop WJ, Gansevoort RT, Vasan RS, Gardin JM, Levy D, Gottdiener JS, de Boer RA and Larson MG. Predicting Heart Failure With Preserved and Reduced Ejection Fraction: The International Collaboration on Heart Failure Subtypes. *Circulation: Heart Failure*. 2016;9(6).

97. Hoffmann U, Massaro JM, D'Agostino RB, Sr., Kathiresan S, Fox CS and O'Donnell CJ. Cardiovascular Event Prediction and Risk Reclassification by Coronary, Aortic, and Valvular Calcification in the Framingham Heart Study. *Journal of the American Heart Association*. 2016;5.

98. Horne BD, Anderson JL, Muhlestein JB, Ridker PM and Paynter NP. Complete blood count risk score and its components, including RDW, are associated with mortality in the JUPITER trial. *Eur J Prev Cardiol*. 2015;22:519-526.

99. Hoshide S, Yano Y, Haimoto H, Yamagiwa K, Uchiba K, Nagasaka S, Matsui Y, Nakamura A, Fukutomi M, Eguchi K, Ishikawa J, Kario K and Group JHS. Morning and Evening Home Blood Pressure and Risks of Incident Stroke and Coronary Artery Disease in the Japanese General Practice Population: The Japan Morning Surge-Home Blood Pressure Study. *Hypertension*. 2016;68:54-61.

100. Howard G, McClure LA, Moy CS, Howard VJ, Judd SE, Yuan Y, Long DL, Muntner P, Safford MM and Kleindorfer DO. Self-Reported Stroke Risk Stratification: Reasons for Geographic and Racial Differences in Stroke Study. *Stroke*. 2017;48:1737-1743.

101. Htun NM, Magliano DJ, Zhang ZY, Lyons J, Petit T, Nkuipou-Kenfack E, Ramirez-Torres A, von Zur Muhlen C, Maahs D, Schanstra JP, Pontillo C, Pejchinovski M, Snell-Bergeon JK, Delles C, Mischak H, Staessen JA, Shaw JE, Koeck T and Peter K. Prediction of acute coronary syndromes by urinary proteome analysis. *PLoS One*. 2017;12:e0172036.

102. Hu G, Root M and Duncan AW. Adding multiple risk factors improves Framingham coronary heart disease risk scores. *Vasc Health Risk Manag*. 2014;10:557-62.

103. Hwang YC, Park CY, Ahn HY and Cho NH. Prediction of future development of cardiovascular disease with an equation to estimate apolipoprotein B: A community-based cohort study. *Medicine*. 2016;95:e3644.

104. Inzhakova G, Zhou H, Morris M, Early MI, Xiang AH, Jacobsen SJ and Derose SF. Potential of Risk-Based Population Guidelines to Reduce Cardiovascular Risk in a Large Integrated Health System. *Am J Manag Care*. 2016;22:E161-+.

105. Iribarren C, Chandra M, Rana JS, Hlatky MA, Fortmann SP, Quertermous T and Go AS. High-sensitivity cardiac troponin I and incident coronary heart disease among asymptomatic older adults. *Heart*. 2016;102:1177-82.

106. Iribarren C, Lu M, Jorgenson E, Martinez M, Lluis-Ganella C, Subirana I, Salas E and Elosua R. Clinical Utility of Multimarker Genetic Risk Scores for Prediction of Incident Coronary Heart Disease: A Cohort Study Among Over 51 Thousand Individuals of European Ancestry. *Circ Cardiovasc Genet*. 2016;9:531-540.

107. Israel A, Kivity S, Sidi Y, Segev S, Berkovitch A, Klempfner R, Lavi B, Goldenberg I and Maor E. Use of exercise capacity to improve SCORE risk prediction model in asymptomatic adults. *Eur Heart J*. 2016;37:2300-6.

108. Jairam PM, de Jong PA, Mali WP, Isgum I, van der Graaf Y and study-group P. Cardiovascular disease prediction: do pulmonary disease-related chest CT features have added value? *Eur Radiol*. 2015;25:1646-54.

109. Jdanov DA, Deev AD, Jasilionis D, Shalnova SA, Shkolnikova MA and Shkolnikov VM. Recalibration of the SCORE risk chart for the Russian population. *Eur J Epidemiol*. 2014;29:621-628.

110. Jee SH, Jang Y, Oh DJ, Oh BH, Lee SH, Park SW, Seung KB, Mok Y, Jung KJ, Kimm H, Yun YD, Baek SJ, Lee DC, Choi SH, Kim MJ, Sung J, Cho B, Kim ES, Yu BY, Lee TY, Kim JS, Lee YJ, Oh JK, Kim SH, Park JK, Koh SB, Park SB, Lee SY, Yoo CI, Kim MC, Kim HK, Park JS, Kim HC, Lee GJ and Woodward M. A coronary heart disease prediction model: the Korean Heart Study. *BMJ Open*. 2014;4:e005025.

111. Johansson JK, Puukka PJ, Niiranen TJ, Varis J, Peltonen M, Salomaa V and Jula AM. Health 2000 score - development and validation of a novel cardiovascular risk score. *Ann Med*. 2016;48:403-409.

112. Jorstad HT, Boekholdt SM, Wareham NJ, Khaw KT and Peters RJ. The Dutch SCORE-based risk charts seriously underestimate the risk of cardiovascular disease. *Netherlands Heart Journal*. 2017;25:173-180.

113. Jorstad HT, Colkesen EB, Minneboo M, Peters RJ, Boekholdt SM, Tijssen JG, Wareham NJ and Khaw KT. The Systematic COronary Risk Evaluation (SCORE) in a large UK population: 10-year follow-up in the EPIC-Norfolk prospective population study. *Eur J Prev Cardiol*. 2015;22:119-26.

114. Jung CH, Lee MJ, Kang YM, Yang DH, Kang JW, Kim EH, Park DW, Park JY, Kim HK and Lee WJ. 2013 ACC/AHA versus 2004 NECP ATP III Guidelines in the Assignment of Statin Treatment in a Korean Population with Subclinical Coronary Atherosclerosis. *PLoS ONE [Electronic Resource]*. 2015;10:e0137478.

115. Jung KJ, Jang Y, Oh DJ, Oh BH, Lee SH, Park SW, Seung KB, Kim HK, Yun YD, Choi SH, Sung J, Lee TY, Kim SH, Koh SB, Kim MC, Chang Kim H, Kimm H, Nam C, Park S and Jee SH. The ACC/AHA 2013 pooled cohort equations compared to a Korean Risk Prediction Model for atherosclerotic cardiovascular disease. *Atherosclerosis*. 2015;242:367-75.

116. Kaess BM, de Las Heras Gala T, Zierer A, Meisinger C, Wahl S, Peters A, Todd J, Herder C, Huth C, Thorand B and Koenig W. Ultra-sensitive troponin I is an independent predictor of incident coronary heart disease in the general population. *Eur J Epidemiol*. 2017;32:583-591.

117. Kalsch H, Lehmann N, Mahabadi AA, Bauer M, Kara K, Huppe P, Moebus S, Mohlenkamp S, Dragano N, Schmermund A, Stang A, Jockel KH, Erbel R and Investigator Group of the Heinz Nixdorf Recall S. Beyond Framingham risk factors and coronary calcification: does aortic valve calcification improve risk prediction? The Heinz Nixdorf Recall Study. *Heart*. 2014;100:930-7.

118. Kanjilal S, Rao VS, Mukherjee M, Natesha BK, Renuka KS, Sibi K, Iyengar SS and Kakkar VV. Application of cardiovascular disease risk prediction models and the relevance of novel biomarkers to risk stratification in Asian Indians. *Vasc Health Risk Manag*. 2008;4:199-211.

119. Karas MG, Devereux RB, Wiebers DO, Whisnant JP, Best LG, Lee ET, Howard BV, Roman MJ, Umans JG and Kizer JR. Incremental value of biochemical and echocardiographic measures in prediction of ischemic stroke: the Strong Heart Study. *Stroke*. 2012;43:720-6.

120. Karjalainen T, Adiels M, Bjorck L, Cooney MT, Graham I, Perk J, Rosengren A, Soderberg S and Eliasson M. An evaluation of the performance of SCORE Sweden 2015 in estimating cardiovascular risk: The Northern Sweden MONICA Study 1999-2014. *Eur J Prev Cardiol*. 2017;24:103-110.

121. Karmali KN, Ning H, Goff DC and Lloyd-Jones DM. Identifying Individuals at Risk for Cardiovascular Events Across the Spectrum of Blood Pressure Levels. *Journal of the American Heart Association*. 2015;4:e002126.

122. Kavousi M, Desai CS, Ayers C, Blumenthal RS, Budoff MJ, Mahabadi AA, Ikram MA, van der Lugt A, Hofman A, Erbel R, Khera A, Geisel MH, Jockel KH, Lehmann N, Hoffmann U, O'Donnell CJ, Massaro JM, Liu K, Mohlenkamp S, Ning H, Franco OH and Greenland P. Prevalence and Prognostic Implications of Coronary Artery Calcification in Low-Risk Women: A Meta-analysis. *JAMA*. 2016;316:2126-2134.

123. Kavousi M, Leening MJ, Nanchen D, Greenland P, Graham IM, Steyerberg EW, Ikram MA, Stricker BH, Hofman A and Franco OH. Comparison of application of the ACC/AHA guidelines, Adult Treatment Panel III guidelines, and European Society of Cardiology guidelines for cardiovascular disease prevention in a European cohort. *JAMA*. 2014;311:1416-23.

124. Khalili D, Asgari S, Hadaegh F, Steyerberg EW, Rahimi K, Fahimfar N and Azizi F. A new approach to test validity and clinical usefulness of the 2013 ACC/AHA guideline on statin therapy: A population-based study. *Int J Cardiol*. 2015;184:587-94.

125. Kim TH, Choi HS, Bae JC, Moon JH, Kim HK, Choi SH, Lim S, Park DJ, Park KS, Jang HC, Lee MK, Cho NH and Park YJ. Subclinical hypothyroidism in addition to common risk scores for prediction of cardiovascular disease: a 10-year community-based cohort study. *Eur J Endocrinol*. 2014;171:649-57.

126. Konety SH, Koene RJ, Norby FL, Wilsdon T, Alonso A, Siscovick D, Sotoodehnia N, Gottdiener J, Fox ER, Chen LY, Adabag S and Folsom AR. Echocardiographic Predictors of Sudden Cardiac Death: The Atherosclerosis Risk in Communities Study and Cardiovascular Health Study. *Circ Cardiovasc Imaging*. 2016;9.

127. Konfino J, Fernandez A, Penko J, Mason A, Martinez E, Coxson P, Heller D, Moran A, Bibbins-Domingo K, Perez-Stable EJ and Mejia R. Comparing Strategies for Lipid Lowering in Argentina: An Analysis from the CVD Policy Model-Argentina. *J Gen Intern Med*. 2017;32:524-533.

128. Kovalchik SA and Pfeiffer RM. Population-based absolute risk estimation with survey data. *Lifetime Data Anal*. 2014;20:252-75.

129. Kreger BE, Cupples LA and Kannel WB. The electrocardiogram in prediction of sudden death: Framingham Study experience. *Am Heart J*. 1987;113:377-82.

130. Kumarathurai P, Mouridsen MR, Mattsson N, Larsen BS, Nielsen OW, Gerds TA and Sajadieh A. Atrial ectopy and N-terminal pro-B-type natriuretic peptide as predictors of atrial fibrillation: a population-based cohort study. *Europace*. 2017;19:364-370.

131. Kunutsor SK, Bakker SJ, Kootstra-Ros JE, Gansevoort RT and Dullaart RP. Circulating gamma glutamyltransferase and prediction of cardiovascular disease. *Atherosclerosis*. 2015;238:356-64.

132. Kunutsor SK, Bakker SJ, Kootstra-Ros JE, Gansevoort RT, Gregson J and Dullaart RP. Serum Alkaline Phosphatase and Risk of Incident Cardiovascular Disease: Interrelationship with High Sensitivity C-Reactive Protein. *PLoS One*. 2015;10:e0132822.

133. Kusmana D. The influence of smoking cessation, regular physical exercise and/or physical activity on survival: A 13 years cohort study of the Indonesian population in Jakarta. *Medical Journal of Indonesia*. 2002;11:230-242.

134. Langley SR, Willeit K, Didangelos A, Matic LP, Skroblin P, Barallobre-Barreiro J, Lengquist M, Rungger G, Kapustin A, Kedenko L, Molenaar C, Lu R, Barwari T, Suna G, Yin X, Iglseder B, Paulweber B, Willeit P, Shalhoub J, Pasterkamp G, Davies AH, Monaco C, Hedin U, Shanahan CM, Willeit J, Kiechl S and Mayr M. Extracellular matrix proteomics identifies molecular signature of symptomatic carotid plaques. *J Clin Invest*. 2017;127:1546-1560.

135. Lee CH, Woo YC, Lam JK, Fong CH, Cheung BM, Lam KS and Tan KC. Validation of the Pooled Cohort equations in a long-term cohort study of Hong Kong Chinese. *J Clin Lipidol*. 2015;9:640-6 e2.

136. Lee JW, Hur J, Choi SI, Chun EJ, Kang JW, Jin GY, Kim EY, Yong HS, Kang EJ, Han K, Lee HS and Choi BW. Incremental prognostic value of computed tomography in stroke: rationale and design of the IMPACTS study. *Int J Cardiovasc Imaging*. 2016;32 Suppl 1:83-9.

137. Lin YH, Glei D, Weinstein M, Wu SI and Chien KL. Additive value of interleukin-6 and C-reactive protein in risk prediction for all-cause and cardiovascular mortality among a representative adult cohort in Taiwan. *J Formos Med Assoc*. 2017;116:982-992.

138. Lindberg S, Jensen JS, Mogelvang R, Pedersen SH, Galatius S, Flyvbjerg A and Magnusson NE. Plasma Neutrophil Gelatinase-Associated Lipocalinin in the General Population Association With Inflammation and Prognosis. *Arterioscl Throm Vas*. 2014;34:2135-2142.

139. Liu L, Tang Z, Li X, Luo Y, Guo J, Li H, Liu X, Tao L, Yan A and Guo X. A novel risk score to the prediction of 10-year risk for coronary artery disease among the elderly in Beijing based on competing risk model. *Medicine (United States)*. 2016;95.

140. Lluis-Ganella C, Subirana I, Lucas G, Tomas M, Munoz D, Senti M, Salas E, Sala J, Ramos R, Ordovas JM, Marrugat J and Elosua R. Assessment of the value of a genetic risk score in improving the estimation of coronary risk. *Atherosclerosis*. 2012;222:456-63.

141. Lopez-Suarez A, Bascunana-Quirell A, Beltran-Robles M, Elvira-Gonzalez J, Fernandez-Palacin F, Barroso-Casamitjana E and Solino-Ocana I. Metabolic syndrome does not improve the prediction of 5-year cardiovascular disease and total mortality over standard risk markers. Prospective population based study. *Medicine*. 2014;93:e212.

142. Lyngbaek S, Marott JL, Sehestedt T, Hansen TW, Olsen MH, Andersen O, Linneberg A, Haugaard SB, Eugen-Olsen J, Hansen PR and Jeppesen J. Cardiovascular risk prediction in the general population with use of suPAR, CRP, and Framingham Risk Score. *Int J Cardiol*. 2013;167:2904-11.

143. Mahabadi AA, Berg MH, Lehmann N, Kalsch H, Bauer M, Kara K, Dragano N, Moebus S, Jockel KH, Erbel R and Mohlenkamp S. Association of epicardial fat with cardiovascular risk factors and incident myocardial infarction in the general population: the Heinz Nixdorf Recall Study. *J Am Coll Cardiol*. 2013;61:1388-95.

144. Mahabadi AA, Lehmann N, Mohlenkamp S, Pundt N, Dykun I, Roggenbuck U, Moebus S, Jockel KH, Erbel R, Kalsch H and Heinz Nixdorf Investigative G. Noncoronary Measures Enhance the Predictive Value of Cardiac CT Above Traditional Risk Factors and CAC Score in the General Population. *JACC Cardiovasc Imaging*. 2016;9:1177-1185.

145. Manuel DG, Tuna M, Perez R, Tanuseputro P, Hennessy D, Bennett C, Rosella L, Sanmartin C, van Walraven C and Tu JV. Predicting Stroke Risk Based on Health Behaviours: Development of the Stroke Population Risk Tool (SPoRT). *PLoS One*. 2015;10:e0143342.

146. Marino M, Li Y, Pencina MJ, D'Agostino RB, Sr., Berkman LF and Buxton OM. Quantifying cardiometabolic risk using modifiable non-self-reported risk factors. *Am J Prev Med*. 2014;47:131-40.

147. Marrugat J, Subirana I, Ramos R, Vila J, Marin-Ibanez A, Guembe MJ, Rigo F, Tormo Diaz MJ, Moreno-Iribas C, Cabre JJ, Segura A, Baena-Diez JM, de la Camara AG, Lapetra J, Grau M, Quesada M, Medrano MJ, Gonzalez Diego P, Frontera G, Gavrila D, Aicua EA, Basora J, Garcia JM, Garcia-Lareo M, Gutierrez JA, Mayoral E, Sala J, D'Agostino R, Elosua R and Investigators F. Derivation and validation of a set of 10-year cardiovascular risk predictive functions in Spain: the FRESCO Study. *Prev Med*. 2014;61:66-74.

148. Matsushita K, Sang Y, Ballew SH, Astor BC, Hoogeveen RC, Solomon SD, Ballantyne CM, Woodward M and Coresh J. Cardiac and kidney markers for cardiovascular prediction in individuals with chronic kidney disease: the Atherosclerosis Risk in Communities study. *Arterioscler Thromb Vasc Biol*. 2014;34:1770-7.

149. McClelland RL, Jorgensen NW, Budoff M, Blaha MJ, Post WS, Kronmal RA, Bild DE, Shea S, Liu K, Watson KE, Folsom AR, Khera A, Ayers C, Mahabadi AA, Lehmann N, Jockel KH, Moebus S, Carr JJ, Erbel R and Burke GL. 10-Year Coronary Heart Disease Risk Prediction Using Coronary Artery Calcium and Traditional Risk Factors: Derivation in the MESA (Multi-Ethnic Study of Atherosclerosis) With Validation in the HNR (Heinz Nixdorf Recall) Study and the DHS (Dallas Heart Study). *J Am Coll Cardiol*. 2015;66:1643-53.

150. McClure LA, Kleindorfer DO, Kissela BM, Cushman M, Soliman EZ and Howard G. Assessing the performance of the Framingham Stroke Risk Score in the reasons for geographic and racial differences in stroke cohort. *Stroke*. 2014;45:1716-20.

151. McNeil JJ, Peeters A, Liew D, Lim S and Vos T. A model for predicting the future incidence of coronary heart disease within percentiles of coronary heart disease risk. *J Cardiovasc Risk*. 2001;8:31-7.

152. Mody P, Joshi PH, Khera A, Ayers CR and Rohatgi A. Beyond Coronary Calcification, Family History, and C-Reactive Protein: Cholesterol Efflux Capacity and Cardiovascular Risk Prediction. *J Am Coll Cardiol*. 2016;67:2480-7.

153. Morris RW, Cooper JA, Shah T, Wong A, Drenos F, Engmann J, McLachlan S, Jefferis B, Dale C, Hardy R, Kuh D, Ben-Shlomo Y, Wannamethee SG, Whincup PH, Casas JP, Kivimaki M, Kumari M, Talmud PJ, Price JF, Dudbridge F, Hingorani AD, Humphries SE and Consortium U. Marginal role for 53 common genetic variants in cardiovascular disease prediction. *Heart*. 2016;102:1640-7.

154. Mortensen MB, Afzal S, Nordestgaard BG and Falk E. The high-density lipoprotein-adjusted SCORE model worsens SCORE-based risk classification in a contemporary population of 30,824 Europeans: the Copenhagen General Population Study. *Eur Heart J*. 2015;36:2446-53.

155. Mortensen MB, Nordestgaard BG, Afzal S and Falk E. ACC/AHA guidelines superior to ESC/EAS guidelines for primary prevention with statins in non-diabetic Europeans: the Copenhagen General Population Study. *Eur Heart J*. 2017;38:586-594.

156. Muntner P, Colantonio LD, Cushman M, Goff DC, Jr., Howard G, Howard VJ, Kissela B, Levitan EB, Lloyd-Jones DM and Safford MM. Validation of the atherosclerotic cardiovascular disease Pooled Cohort risk equations. *JAMA*. 2014;311:1406-15.

157. Nambi V, Liu X, Chambless LE, de Lemos JA, Virani SS, Agarwal S, Boerwinkle E, Hoogeveen RC, Aguilar D, Astor BC, Srinivas PR, Deswal A, Mosley TH, Coresh J, Folsom AR, Heiss G and Ballantyne CM. Troponin T and N-terminal pro-B-type natriuretic peptide: a biomarker approach to predict heart failure risk--the atherosclerosis risk in communities study. *Clin Chem*. 2013;59:1802-10.

158. Nargesi AA, Heidari B, Esteghamati S, Hafezi-Nejad N, Sheikhbahaei S, Pajouhi A, Nakhjavani M and Esteghamati A. Contribution of vitamin D deficiency to the risk of coronary heart disease in subjects with essential hypertension. *Atherosclerosis*. 2016;244:165-71.

159. Nauman J, Nes BM, Lavie CJ, Jackson AS, Sui X, Coombes JS, Blair SN and Wisloff U. Prediction of Cardiovascular Mortality by Estimated Cardiorespiratory Fitness Independent of Traditional Risk Factors: The HUNT Study. *Mayo Clin Proc*. 2017;92:218-227.

160. Ndumele CE, Matsushita K, Sang Y, Lazo M, Agarwal SK, Nambi V, Deswal A, Blumenthal RS, Ballantyne CM, Coresh J and Selvin E. N-Terminal Pro-Brain Natriuretic Peptide and Heart Failure Risk Among Individuals With and Without Obesity: The Atherosclerosis Risk in Communities (ARIC) Study. *Circulation*. 2016;133:631-8.

161. Nielsen JB, Graff C, Rasmussen PV, Pietersen A, Lind B, Olesen MS, Struijk JJ, Haunso S, Svendsen JH, Kober L, Gerds TA and Holst AG. Risk prediction of cardiovascular death based on the QTc interval: evaluating age and gender differences in a large primary care population. *Eur Heart J*. 2014;35:1335-44.

162. Ninomiya T, Kojima I, Doi Y, Fukuhara M, Hirakawa Y, Hata J, Kitazono T and Kiyohara Y. Brachial-ankle pulse wave velocity predicts the development of cardiovascular disease in a general Japanese population: the Hisayama Study. *J Hypertens*. 2013;31:477-83; discussion 483.

163. Nishimura K, Okamura T, Watanabe M, Nakai M, Takegami M, Higashiyama A, Kokubo Y, Okayama A and Miyamoto Y. Predicting coronary heart disease using risk factor categories for a Japanese urban population, and comparison with the framingham risk score: the suita study. *J Atheroscler Thromb*. 2014;21:784-98.

164. Nobel L, Mayo NE, Hanley J, Nadeau L and Daskalopoulou SS. MyRisk_Stroke Calculator: A Personalized Stroke Risk Assessment Tool for the General Population. *J Clin Neurol*. 2014;10:1-9.

165. Ogunwale AN, Morrison AC, Sun W, Dodge RC, Virani SS, Taylor A, Gottesman RF, Yang E, Wei P, McEvoy JW, Heiss G, Boerwinkle E, Ballantyne CM and Nambi V. The impact of multiple single day blood pressure readings on cardiovascular risk estimation: The Atherosclerosis Risk in Communities study. *Eur J Prev Cardiol*. 2016;23:1529-36.

166. Okwuosa TM, Soliman EZ, Lopez F, Williams KA, Alonso A and Ferdinand KC. Left ventricular hypertrophy and cardiovascular disease risk prediction and reclassification in blacks and whites: the Atherosclerosis Risk in Communities Study. *Am Heart J*. 2015;169:155-61 e5.

167. Onat A, Can G, Kaya A, Keskin M, Hayiroglu MI and Yuksel H. Algorithm for predicting CHD death risk in Turkish adults: conventional factors contribute only moderately in women. *Anatol J Cardiol*. 2017;17:436-444.

168. Paixao AR, Ayers CR, El Sabbagh A, Sanghavi M, Berry JD, Rohatgi A, Kumbhani DJ, McGuire DK, Das SR, de Lemos JA and Khera A. Coronary Artery Calcium Improves Risk Classification in Younger Populations. *JACC Cardiovasc Imaging*. 2015;8:1285-93.

169. Panagiotakos DB, Georgousopoulou EN, Fitzgerald AP, Pitsavos C and Stefanadis C. Validation of the HellenicSCORE (a Calibration of the ESC SCORE Project) Regarding 10-Year Risk of Fatal Cardiovascular Disease in Greece. *Hell J Cardiol*. 2015;56:302-308.

170. Parikh NI, Jeppson RP, Berger JS, Eaton CB, Kroenke CH, LeBlanc ES, Lewis CE, Loucks EB, Parker DR, Rillamas-Sun E, Ryckman KK, Waring ME, Schenken RS, Johnson KC, Edstedt-Bonamy AK, Allison MA and Howard BV. Reproductive Risk Factors and Coronary Heart Disease in the Women's Health Initiative Observational Study. *Circulation*. 2016;133:2149-58.

171. Park GM, Han S, Kim SH, Jo MW, Her SH, Lee JB, Lee MS, Kim HC, Ahn JM, Lee SW, Kim YH, Kim BJ, Koh JM, Kim HK, Choe J, Park SW and Park SJ. Model for assessing cardiovascular risk in a Korean population. *Circ Cardiovasc Qual Outcomes*. 2014;7:944-51.

172. Parmar P, Krishnamurthi R, Ikram MA, Hofman A, Mirza SS, Varakin Y, Kravchenko M, Piradov M, Thrift AG, Norrving B, Wang W, Mandal DK, Barker-Collo S, Sahathevan R, Davis S, Saposnik G, Kivipelto M, Sindi S, Bornstein NM, Giroud M, Bejot Y, Brainin M, Poulton R, Narayan KM, Correia M, Freire A, Kokubo Y, Wiebers D, Mensah G, BinDhim NF, Barber PA, Pandian JD, Hankey GJ, Mehndiratta MM, Azhagammal S, Ibrahim NM, Abbott M, Rush E, Hume P, Hussein T, Bhattacharjee R, Purohit M, Feigin VL and Stroke Riskometer TMCWG. The Stroke Riskometer(TM) App: validation of a data collection tool and stroke risk predictor. *Int J Stroke*. 2015;10:231-44.

173. Paynter NP, Crainiceanu CM, Sharrett AR, Chambless LE and Coresh J. Effect of correcting for long-term variation in major coronary heart disease risk factors: relative hazard estimation and risk prediction in the Atherosclerosis Risk in Communities Study. *Ann Epidemiol*. 2012;22:191-7.

174. Paynter NP, LaMonte MJ, Manson JE, Martin LW, Phillips LS, Ridker PM, Robinson JG and Cook NR. Comparison of lifestyle-based and traditional cardiovascular disease prediction in a multiethnic cohort of nonsmoking women. *Circulation*. 2014;130:1466-73.

175. Pencina MJ, D'Agostino RB, Zdrojewski T, Williams K, Thanassoulis G, Furberg CD, Peterson ED, Vasan RS and Sniderman AD. Apolipoprotein B improves risk assessment of future coronary heart disease in the Framingham Heart Study beyond LDL-C and non-HDL-C. *Eur J Prev Cardiol*. 2015;22:1321-7.

176. Perez HA, Garcia NH, Spence JD and Armando LJ. Adding carotid total plaque area to the Framingham risk score improves cardiovascular risk classification. *Arch Med Sci*. 2016;12:513-20.

177. Piotrowski W, Waskiewicz A and Cicha-Mikolajczyk A. Global cardiovascular mortality risk in the adult Polish population: prospective assessment of the cohorts studied in multicentre national WOBASZ and WOBASZ Senior studies. *Kardiologia Polska*. 2016;74:262-73.

178. Polak JF, Szklo M and O'Leary DH. Associations of Coronary Heart Disease with Common Carotid Artery Near and Far Wall Intima-Media Thickness: The Multi-Ethnic Study of Atherosclerosis. *J Am Soc Echocardiogr*. 2015;28:1114-21.

179. Polenz GF, Leiria TL, Essebag V, Kruse ML, Pires LM, Nogueira TB, Guimaraes RB, Santanna RT and GG DEL. CHA2 DS2 VASc Score as a Predictor of Cardiovascular Events in Ambulatory Patients without Atrial Fibrillation. *Pacing Clin Electrophysiol*. 2015;38:1412-7.

180. Qureshi WT, Michos ED, Flueckiger P, Blaha M, Sandfort V, Herrington DM, Burke G and Yeboah J. Impact of Replacing the Pooled Cohort Equation With Other Cardiovascular Disease Risk Scores on Atherosclerotic Cardiovascular Disease Risk Assessment (from the Multi-Ethnic Study of Atherosclerosis [MESA]). *American Journal of Cardiology*. 2016;118:691-6.

181. Raggi P, Cooil B and Callister TQ. Use of electron beam tomography data to develop models for prediction of hard coronary events. *Am Heart J*. 2001;141:375-82.

182. Raghu A, Praveen D, Peiris D, Tarassenko L and Clifford G. Implications of Cardiovascular Disease Risk Assessment Using the WHO/ISH Risk Prediction Charts in Rural India. *PLoS One*. 2015;10:e0133618.

183. Rana JS, Tabada GH, Solomon MD, Lo JC, Jaffe MG, Sung SH, Ballantyne CM and Go AS. Accuracy of the Atherosclerotic Cardiovascular Risk Equation in a Large Contemporary, Multiethnic Population. *J Am Coll Cardiol*. 2016;67:2118-2130.

184. Romanens M, Ackermann F, Sudano I, Szucs T and Spence JD. Arterial age as a substitute for chronological age in the AGLA risk function could improve coronary risk prediction. *Swiss Med Wkly*. 2014;144:w13967.

185. Rucker V, Keil U, Fitzgerald AP, Malzahn U, Prugger C, Ertl G, Heuschmann PU and Neuhauser H. Predicting 10-Year Risk of Fatal Cardiovascular Disease in Germany: An Update Based on the SCORE-Deutschland Risk Charts. *PLoS One*. 2016;11:e0162188.

186. Sabayan B, Gussekloo J, de Ruijter W, Westendorp RG and de Craen AJ. Framingham stroke risk score and cognitive impairment for predicting first-time stroke in the oldest old. *Stroke*. 2013;44:1866-71.

187. Salim A, Tai ES, Tan VY, Welsh AH, Liew R, Naidoo N, Wu Y, Yuan JM, Koh WP and van Dam RM. C-reactive protein and serum creatinine, but not haemoglobin A1c, are independent predictors of coronary heart disease risk in non-diabetic Chinese. *Eur J Prev Cardiolog*. 2016;23:1339-49.

188. Sanchez-Inigo L, Navarro-Gonzalez D, Fernandez-Montero A, Pastrana-Delgado J and Martinez JA. The TyG index may predict the development of cardiovascular events. *European Journal of Clinical Investigation*. 2016;46:189-97.

189. Sardarinia M, Ansari R, Azizi F, Hadaegh F and Bozorgmanesh M. Mortality prediction of a body shape index versus traditional anthropometric measures in an Iranian population: Tehran Lipid and Glucose Study. *Nutrition*. 2017;33:105-112.

190. Sawano M, Kohsaka S, Okamura T, Inohara T, Sugiyama D, Watanabe M, Nakamura Y, Higashiyama A, Kadota A, Okud N, Murakami Y, Ohkubo T, Fujiyoshi A, Miura K, Okayama A, Ueshima H, National Integrated Project for Prospective Observation of Non-Communicable D and its Trends in the Aged Research G. Validation of the european SCORE risk chart in the healthy middle-aged Japanese. *Atherosclerosis*. 2016;252:116-21.

191. Schiopu A, Bengtsson E, Goncalves I, Nilsson J, Fredrikson GN and Bjorkbacka H. Associations Between Macrophage Colony-Stimulating Factor and Monocyte Chemotactic Protein 1 in Plasma and First-Time Coronary Events: A Nested Case-Control Study. *Journal of the American Heart Association*. 2016;5.

192. Schnohr P, Marott JL, Kristensen TS, Gyntelberg F, Gronbaek M, Lange P, Jensen MT, Jensen GB and Prescott E. Ranking of psychosocial and traditional risk factors by importance for coronary heart disease: the Copenhagen City Heart Study. *Eur Heart J*. 2015;36:1385-93.

193. Sehestedt T, Jeppesen J, Hansen TW, Rasmussen S, Wachtell K, Ibsen H, Torp-Pedersen C and Olsen MH. Can ambulatory blood pressure measurements substitute assessment of subclinical cardiovascular damage? *J Hypertens*. 2012;30:513-21.

194. Seidelmann SB, Claggett B, Bravo PE, Gupta A, Farhad H, Klein BE, Klein R, Di Carli M and Solomon SD. Retinal Vessel Calibers in Predicting Long-Term Cardiovascular Outcomes: The Atherosclerosis Risk in Communities Study. *Circulation*. 2016;134:1328-1338.

195. Selmer R, Igland J, Ariansen I, Tverdal A, Njolstad I, Furu K, Tell GS and Klemsdal TO. NORRISK 2: A Norwegian risk model for acute cerebral stroke and myocardial infarction. *Eur J Prev Cardiol*. 2017;24:773-782.

196. Selvarajah S, Kaur G, Haniff J, Cheong KC, Hiong TG, van der Graaf Y and Bots ML. Comparison of the Framingham Risk Score, SCORE and WHO/ISH cardiovascular risk prediction models in an Asian population. *Int J Cardiol*. 2014;176:211-8.

197. Sepanlou SG, Malekzadeh R, Poustchi H, Sharafkhah M, Ghodsi S, Malekzadeh F, Etemadi A, Pourshams A, Pharoah PD, Abnet CC, Brennan P, Boffetta P, Dawsey SM and Kamangar F. The clinical performance of an office-based risk scoring system for fatal cardiovascular diseases in North-East of Iran. *PLoS One*. 2015;10:e0126779.

198. Shah N, Pahuja M, Pant S, Handa A, Agarwal V, Patel N and Dusaj R. Red cell distribution width and risk of cardiovascular mortality: Insights from National Health and Nutrition Examination Survey (NHANES)-III. *Int J Cardiol*. 2017;232:105-110.

199. Shah N, Parikh V, Patel N, Patel N, Badheka A, Deshmukh A, Rathod A and Lafferty J. Neutrophil lymphocyte ratio significantly improves the Framingham risk score in prediction of coronary heart disease mortality: insights from the National Health and Nutrition Examination Survey-III. *Int J Cardiol*. 2014;171:390-7.

200. Sharma A, Ghatge M, Mundkur L and Vangala RK. Translational informatics approach for identifying the functional molecular communicators linking coronary artery disease, infection and inflammation. *Mol Med Rep*. 2016;13:3904-12.

201. Shoamanesh A, Preis SR, Beiser AS, Kase CS, Wolf PA, Vasan RS, Benjamin EJ, Seshadri S and Romero JR. Circulating biomarkers and incident ischemic stroke in the Framingham Offspring Study. *Neurology*. 2016;87:1206-11.

202. Sim J, Teece L, Dennis MS, Roffe C and Team SSS. Validation and Recalibration of Two Multivariable Prognostic Models for Survival and Independence in Acute Stroke. *Plos One*. 2016;11.

203. Solbu MD, Toft I, Lochen ML, Mathiesen EB, Eriksen BO, Melsom T, Njolstad I, Wilsgaard T and Jenssen TG. N-Acetyl-beta-D-Glucosaminidase Does Not Enhance Prediction of Cardiovascular or All-Cause Mortality by Albuminuria in a Low-Risk Population. *J Am Soc Nephrol*. 2016;27:533-42.

204. Sonneveld MA, de Maat MP, Portegies ML, Kavousi M, Hofman A, Turecek PL, Rottensteiner H, Scheiflinger F, Koudstaal PJ, Ikram MA and Leebeek FW. Low ADAMTS13 activity is associated with an increased risk of ischemic stroke. *Blood*. 2015;126:2739-46.

205. Stam-Slob MC, Visseren FLJ, Jukema JW, van der Graaf Y, Poulter NR, Gupta A, Sattar N, Macfarlane PW, Kearney PM, de Craen AJM and Trompet S. Personalized absolute benefit of statin treatment for primary or secondary prevention of vascular disease in individual elderly patients. *Clin Res Cardiol*. 2017;106:58-68.

206. Tada H, Melander O, Louie JZ, Catanese JJ, Rowland CM, Devlin JJ, Kathiresan S and Shiffman D. Risk prediction by genetic risk scores for coronary heart disease is independent of self-reported family history. *Eur Heart J*. 2016;37:561-7.

207. Tang WH, Topol EJ, Fan Y, Wu Y, Cho L, Stevenson C, Ellis SG and Hazen SL. Prognostic value of estimated functional capacity incremental to cardiac biomarkers in stable cardiac patients. *Journal of the American Heart Association*. 2014;3:e000960.

208. Thompson SG, Pyke SD and Wood DA. Using a coronary risk score for screening and intervention in general practice. British Family Heart Study. *J Cardiovasc Risk*. 1996;3:301-6.

209. Tillin T, Hughes AD, Whincup P, Mayet J, Sattar N, McKeigue PM, Chaturvedi N and Group SS. Ethnicity and prediction of cardiovascular disease: performance of QRISK2 and Framingham scores in a U.K. tri-ethnic prospective cohort study (SABRE--Southall And Brent REvisited). *Heart*. 2014;100:60-7.

210. Valenti V, B OH, Heo R, Cho I, Schulman-Marcus J, Gransar H, Truong QA, Shaw LJ, Knapper J, Kelkar AA, Sandesara P, Lin FY, Sciarretta S, Chang HJ, Callister TQ and Min JK. A 15-Year Warranty Period for Asymptomatic Individuals Without Coronary Artery Calcium: A Prospective Follow-Up of 9,715 Individuals. *JACC Cardiovasc Imaging*. 2015;8:900-9.

211. van der Meer MG, van der Graaf Y, Schuit E, Peelen LM, Verschuren WM, Boer JM, Moons KG, Nathoe HM, Appelman Y and van der Schouw YT. Added Value of Female-Specific Factors Beyond Traditional Predictors for Future Cardiovascular Disease. *J Am Coll Cardiol*. 2016;67:2084-6.

212. van Dis I, Geleijnse JM, Boer JM, Kromhout D, Boshuizen H, Grobbee DE, van der Schouw YT and Verschuren WM. Effect of including nonfatal events in cardiovascular risk estimation, illustrated with data from The Netherlands. *Eur J Prev Cardiol*. 2014;21:377-83.

213. van Dis I, Geleijnse JM, Kromhout D, Boer J, Boshuizen H and Verschuren WM. Do obesity and parental history of myocardial infarction improve cardiovascular risk prediction? *Eur J Prev Cardiol*. 2013;20:793-9.

214. van Kempen BJ, Ferket BS, Kavousi M, Leening MJ, Steyerberg EW, Ikram MA, Witteman JC, Hofman A, Franco OH and Hunink MG. Performance of Framingham cardiovascular disease (CVD) predictions in the Rotterdam Study taking into account competing risks and disentangling CVD into coronary heart disease (CHD) and stroke. *Int J Cardiol*. 2014;171:413-8.

215. Vartiainen E, Laatikainen T, Peltonen M and Puska P. Predicting Coronary Heart Disease and Stroke The FINRISK Calculator. *Glob Heart*. 2016;11:213-216.

216. Velescu A, Clara A, Penafiel J, Ramos R, Marti R, Grau M, Degano IR, Marrugat J, Elosua R and Group RS. Adding low ankle brachial index to classical risk factors improves the prediction of major cardiovascular events. The REGICOR study. *Atherosclerosis*. 2015;241:357-63.

217. Venkatesh S, O'Neal WT, Broughton ST, Shah AJ and Soliman EZ. Utility of Normal Findings on Electrocardiogram and Echocardiogram in Subjects >= 65 Years. *American Journal of Cardiology*. 2017;119:856-861.

218. Verbeek R, Sandhu MS, Hovingh GK, Sjouke B, Wareham NJ, Zwinderman AH, Kastelein JJ, Khaw KT, Tsimikas S and Boekholdt SM. Lipoprotein(a) Improves Cardiovascular Risk Prediction Based on Established Risk Algorithms. *J Am Coll Cardiol*. 2017;69:1513-1515.

219. Veronesi G, Giampaoli S, Vanuzzo D, Gianfagna F, Palmieri L, Grassi G, Cesana G and Ferrario MM. Combined use of short-term and long-term cardiovascular risk scores in primary prevention: An assessment of clinical utility. *Journal of Cardiovascular Medicine*. 2017;18:318-324.

220. Veronesi G, Gianfagna F, Giampaoli S, Chambless LE, Grassi G, Cesana G and Ferrario MM. Validity of a long-term cardiovascular disease risk prediction equation for low-incidence populations: the CAMUNI-MATISS Cohorts Collaboration study. *Eur J Prev Cardiol*. 2015;22:1618-25.

221. Veronesi G, Gianfagna F, Giampaoli S, Chambless LE, Mancia G, Cesana G and Ferrario MM. Improving long-term prediction of first cardiovascular event: The contribution of family history of coronary heart disease and social status. *Preventive Medicine*. 2014;64:75-80.

222. Vikhireva O, Broda G, Kubinova R, Malyutina S, Pajak A, Tamosiunas A, Skodova Z, Simonova G, Bobak M and Pikhart H. Does Inclusion of Education and Marital Status Improve SCORE Performance in Central and Eastern Europe and Former Soviet Union? Findings from MONICA and HAPIEE Cohorts. *Plos One*. 2014;9.

223. Vikhireva O, Pajak A, Broda G, Malyutina S, Tamosiunas A, Kubinova R, Simonova G, Skodova Z, Bobak M and Pikhart H. SCORE performance in Central and Eastern Europe and former Soviet Union: MONICA and HAPIEE results. *Eur Heart J*. 2014;35:571-7.

224. Waks JW, Sitlani CM, Soliman EZ, Kabir M, Ghafoori E, Biggs ML, Henrikson CA, Sotoodehnia N, Biering-Sorensen T, Agarwal SK, Siscovick DS, Post WS, Solomon SD, Buxton AE, Josephson ME and Tereshchenko LG. Global Electric Heterogeneity Risk Score for Prediction of Sudden Cardiac Death in the General Population: The Atherosclerosis Risk in Communities (ARIC) and Cardiovascular Health (CHS) Studies. *Circulation*. 2016;133:2222-34.

225. Wang Y, Liu J, Wang W, Wang M, Qi Y, Xie WX, Li Y, Sun JY, Liu J and Zhao D. Lifetime risk of stroke in young-aged andmiddle-aged Chinese population: the Chinese Multi-Provincial Cohort Study. *J Hypertens*. 2016;34:2434-2440.

226. Wassertheil-Smoller S, McGinn A, Allison M, Ca T, Curb D, Eaton C, Hendrix S, Kaplan R, Ko M, Martin LW and Xue X. Improvement in stroke risk prediction: role of C-reactive protein and lipoprotein-associated phospholipase A2 in the women's health initiative. *Int J Stroke*. 2014;9:902-9.

227. Weatherley ND and Jackson PR. The new Sheffield risk and benefit tables for the elderly. *QJM*. 2011;104:3-12.

228. Welsh P, Hart C, Papacosta O, Preiss D, McConnachie A, Murray H, Ramsay S, Upton M, Watt G, Whincup P, Wannamethee G and Sattar N. Prediction of Cardiovascular Disease Risk by Cardiac Biomarkers in 2 United Kingdom Cohort Studies: Does Utility Depend on Risk Thresholds For Treatment? *Hypertension*. 2016;67:309-15.

229. Weng SF, Kai J, Guha IN and Qureshi N. The value of aspartate aminotransferase and alanine aminotransferase in cardiovascular disease risk assessment. *Open Heart*. 2015;2:e000272.

230. Wickramasinghe CD, Ayers CR, Das S, de Lemos JA, Willis BL and Berry JD. Prediction of 30-Year Risk for Cardiovascular Mortality by Fitness and Risk Factor Levels The Cooper Center Longitudinal Study. *Circ-Cardiovasc Qual*. 2014;7:597-602.

231. Willeit P, Kiechl S, Kronenberg F, Witztum JL, Santer P, Mayr M, Xu Q, Mayr A, Willeit J and Tsimikas S. Discrimination and net reclassification of cardiovascular risk with lipoprotein(a): prospective 15-year outcomes in the Bruneck Study. *J Am Coll Cardiol*. 2014;64:851-60.

232. Woodward M. Coronary risk prediction for those with and without diabetes. *European Journal of Cardiovascular Prevention and Rehabilitation*. 2006;13:30-36.

233. Woodward M, Brindle P, Tunstall-Pedoe H and estimation Sgor. Adding social deprivation and family history to cardiovascular risk assessment: the ASSIGN score from the Scottish Heart Health Extended Cohort (SHHEC). *Heart*. 2007;93:172-6.

234. Woznicka-Leskiewicz L, Posadzy-Malaczynska A and Juszkat R. The impact of ankle brachial index and pulse wave velocity on cardiovascular risk according to SCORE and Framingham scales and sex differences. *J Hum Hypertens*. 2015;29:502-10.

235. Wurtz P, Havulinna AS, Soininen P, Tynkkynen T, Prieto-Merino D, Tillin T, Ghorbani A, Artati A, Wang Q, Tiainen M, Kangas AJ, Kettunen J, Kaikkonen J, Mikkila V, Jula A, Kahonen M, Lehtimaki T, Lawlor DA, Gaunt TR, Hughes AD, Sattar N, Illig T, Adamski J, Wang TJ, Perola M, Ripatti S, Vasan RS, Raitakari OT, Gerszten RE, Casas JP, Chaturvedi N, Ala-Korpela M and Salomaa V. Metabolite profiling and cardiovascular event risk: a prospective study of 3 population-based cohorts. *Circulation*. 2015;131:774-85.

236. Yang X, Li J, Hu D, Chen J, Li Y, Huang J, Liu X, Liu F, Cao J, Shen C, Yu L, Lu F, Wu X, Zhao L, Wu X and Gu D. Predicting the 10-Year Risks of Atherosclerotic Cardiovascular Disease in Chinese Population: The China-PAR Project (Prediction for ASCVD Risk in China). *Circulation*. 2016;134:1430-1440.

237. Yatsuya H, Iso H, Li Y, Yamagishi K, Kokubo Y, Saito I, Sawada N, Inoue M and Tsugane S. Development of a Risk Equation for the Incidence of Coronary Artery Disease and Ischemic Stroke for Middle-Aged Japanese- Japan Public Health Center-Based Prospective Study. *Circulation Journal*. 2016;80:1386-95.

238. Yeboah J, Carr JJ, Terry JG, Ding J, Zeb I, Liu S, Nasir K, Post W, Blumenthal RS and Budoff MJ. Computed tomography-derived cardiovascular risk markers, incident cardiovascular events, and all-cause mortality in nondiabetics: the Multi-Ethnic Study of Atherosclerosis. *Eur J Prev Cardiol*. 2014;21:1233-41.

239. Yeboah J, Young R, McClelland RL, Delaney JC, Polonsky TS, Dawood FZ, Blaha MJ, Miedema MD, Sibley CT, Carr JJ, Burke GL, Goff DC, Jr., Psaty BM, Greenland P and Herrington DM. Utility of Nontraditional Risk Markers in Atherosclerotic Cardiovascular Disease Risk Assessment. *J Am Coll Cardiol*. 2016;67:139-147.

240. Yuan Z, Voss EA, DeFalco FJ, Pan G, Ryan PB, Yannicelli D and Nessel C. Risk Prediction for Ischemic Stroke and Transient Ischemic Attack in Patients Without Atrial Fibrillation: A Retrospective Cohort Study. *J Stroke Cerebrovasc Dis*. 2017;26:1721-1731.

241. Yudkin J. Beta-blockers in heart failure. *Diabetic Medicine*. 1999;16:785-7.

242. Zalawadiya SK, Veeranna V, Mallikethi-Reddy S, Bavishi C, Lunagaria A, Kottam A and Afonso L. Uric acid and cardiovascular disease risk reclassification: findings from NHANES III. *Eur J Prev Cardiol*. 2015;22:513-8.

243. Zhang Y, Schottker B, Florath I, Stock C, Butterbach K, Holleczek B, Mons U and Brenner H. Smoking-Associated DNA Methylation Biomarkers and Their Predictive Value for All-Cause and Cardiovascular Mortality. *Environ Health Perspect*. 2016;124:67-74.

244. Zhang Z, Gillespie C, Bowman B and Yang Q. Prediction of atherosclerotic cardiovascular disease mortality in a nationally representative cohort using a set of risk factors from pooled cohort risk equations. *PLoS ONE*. 2017;12.

245. Zhou XH, Wang X, Duncan A, Hu G and Zheng J. Statistical evaluation of adding multiple risk factors improves Framingham stroke risk score. *BMC Med Res Methodol*. 2017;17:58.

246. Ziegelbauer K, Schaefer C, Steinmetz H, Sitzer M and Lorenz MW. Clinical usefulness of carotid ultrasound to improve stroke risk assessment: ten-year results from the Carotid Atherosclerosis Progression Study (CAPS). *Eur J Prev Cardiol*. 2013;20:837-43.
